# Supplementary material for: Mapping density, diversity and species-richness of the Amazon tree flora
Source: Commun Biol. 2023 Nov 8;6:1130. doi: 10.1038/s42003-023-05514-6 (PMC10632362; doi:10.1038/s42003-023-05514-6)
Supplement: Supplementary file 2 — Supplementary Information [file 42003_2023_5514_MOESM2_ESM.pdf]

Mapping density, diversity and species-richness of the Amazon tree flora – supplementary data

Hans ter Steege,1,2 Nigel C.A. Pitman,3 Iêda Leão do Amaral,4 Luiz de Souza Coelho,4 Francisca Dionízia de Almeida Matos,4 Diógenes de Andrade Lima Filho,4 Rafael P. Salomão,5,6 Florian Wittmann,7,8 Carolina V. Castilho,9 Juan Ernesto Guevara,10,11 Marcelo de Jesus Veiga Carim,12 Oliver L. Phillips,13 William E. Magnusson,14 Daniel Sabatier,15 Juan David Cardenas Revilla,4 Jean-François Molino,15 Mariana Victória Irume,4 Maria Pires Martins,4 José Renan da Silva Guimarães,16 José Ferreira Ramos,4 Olaf S. Bánki,1 Maria Teresa Fernandez Piedade,8 Dairon Cárdenas López,17† Domingos de Jesus Rodrigues,18 Layon O. Demarchi,8 Jochen Schöngart,8 Everton José Almeida,19 Luciane Ferreira Barbosa,19 Larissa Cavalheiro,19 Márcia Cléia Vilela dos Santos,19 Bruno Garcia Luize,20 Evlyn Márcia Moraes de Leão Novo,21 Percy Núñez Vargas,22 Thiago Sanna Freire Silva,23 Eduardo Martins Venticinque,24 Angelo Gilberto Manzatto,25 Neidiane Farias Costa Reis,26 John Terborgh,27,28 Katia Regina Casula,26 Euridice N. Honorio Coronado,29,30 Abel Monteagudo Mendoza,22,31 Juan Carlos Montero,32,4 Flávia R.C. Costa,14 Ted R. Feldpausch,33,13 Adriano Costa Quaresma,7,8 Nicolás Castaño Arboleda,17 Charles Eugene Zartman,4 Timothy J. Killeen,34 Beatriz S. Marimon,35 Ben Hur Marimon-Junior,35 Rodolfo Vasquez,31 Bonifacio Mostacedo,36 Rafael L. Assis,37 Chris Baraloto,38 Dário Dantas do Amaral,6 Julien Engel,15,38 Pascal Petronelli,39 Hernán Castellanos,40 Marcelo Brilhante de Medeiros,41 Marcelo Fragomeni Simon,41 Ana Andrade,42 José Luís Camargo,42 William F. Laurance,28 Susan G.W. Laurance,28 Lorena Maniguaje Rincón,4 Juliana Schietti,4 Thaiane R. Sousa,43 Emanuelle de Sousa Farias,44,45 Maria Aparecida Lopes,46 José Leonardo Lima Magalhães,47,48 Henrique Eduardo Mendonça Nascimento,4 Helder Lima de Queiroz,49 Gerardo A. Aymard C.,50 Roel Brienen,13 Pablo R. Stevenson,51 Alejandro Araujo-Murakami,52 Tim R. Baker,13 Bruno Barçante Ladvocat Cintra,53 Yuri Oliveira Feitosa,54 Hugo F. Mogollón,55 Joost F. Duivenvoorden,56 Carlos A. Peres,57 Miles R. Silman,58 Leandro Valle Ferreira,6 José Rafael Lozada,59 James A. Comiskey,60,61 Freddie C. Draper,62 José Julio de Toledo,63 Gabriel Damasco,64 Roosevelt García-Villacorta,65,66 Aline Lopes,67 Alberto Vicentini,14 Fernando Cornejo Valverde,68 Alfonso Alonso,61 Luzmila Arroyo,52 Francisco Dallmeier,61 Vitor H.F. Gomes,69,70 Eliana M. Jimenez,71 David Neill,72 Maria Cristina Peñuela Mora,73 Janaína Costa Noronha,18 Daniel P. P. de Aguiar,74,75 Flávia Rodrigues Barbosa,18 Yennie K. Bredin,76,77 Rainiellen de Sá Carpanedo,18 Fernanda Antunes Carvalho,14,78 Fernanda Coelho de Souza,14,13 Kenneth J. Feeley,79,80 Rogerio Gribel,4 Torbjørn Haugaasen,76 Joseph E. Hawes,81 Marcelo Petratti Pansonato,4,82 Marcos Ríos Paredes,83 Jos Barlow,84 Erika Berenguer,85,84 Izaias Brasil da Silva,86 Maria Julia Ferreira,87 Joice Ferreira,48 Paul V.A. Fine,88 Marcelino Carneiro Guedes,89 Carolina Levis,90 Juan Carlos Licona,32 Boris Eduardo Villa Zegarra,91 Vincent Antoine Vos,92 Carlos Cerón,93 Flávia Machado Durgante,8,7 Émile Fonty,94,15 Terry W. Henkel,95 John Ethan Householder,7 Isau Huamantupa-Chuquimaco,96 Edwin Pos,2,97 Marcos Silveira,98 Juliana Stropp,99 Raquel Thomas,100 Doug Daly,101 Kyle G. Dexter,102,103 William Milliken,104 Guido Pardo Molina,92 Toby Pennington,33,103 Ima Célia Guimarães Vieira,6 Bianca Weiss Albuquerque,8 Wegliane Campelo,63 Alfredo Fuentes,105,106 Bente Klitgaard,107 José Luis Marcelo Pena,108 J. Sebastián Tello,106 Corine Vriesendorp,3 Jerome Chave,109 Anthony Di Fiore,110,111 Renato Richard Hilário,63 Luciana de Oliveira Pereira,33 Juan Fernando Phillips,112 Gonzalo Rivas-Torres,111,113 Tinde R. van Andel,1,114 Patricio von Hildebrand,115 William Balee,116 Edelcilio Marques Barbosa,4 Luiz Carlos de Matos Bonates,4 Hilda Paulette Dávila Doza,83 Ricardo Zárate Gómez,117 Therany Gonzales,118 George Pepe Gallardo Gonzales,83 Bruce Hoffman,119 André Braga Junqueira,120 Yadvinder Malhi,121 Ires Paula de Andrade Miranda,4

Linder Felipe Mozombite Pinto,[83] Adriana Prieto,[122] Agustín Rudas,[122] Ademir R. Ruschel,[48] Natalino Silva,[123] César I.A. Vela,[124] Egleé L. Zent,[125] Stanford Zent,[125] Angela Cano,[51,126] Yrma Andreina Carrero Márquez,[127] Diego F. Correa,[51,128] Janaina Barbosa Pedrosa Costa,[89] Bernardo Monteiro Flores,[90] David Galbraith,[13] Milena Holmgren,[129] Michelle Kalamandeen,[130] Guilherme Lobo,[131] Luis Torres Montenegro,[132] Marcelo Trindade Nascimento,[133] Alexandre A. Oliveira,[82] Maihyra Marina Pombo,[4] Hirma Ramirez-Angulo,[134] Maira Rocha,[8] Veridiana Vizoni Scudeller,[135] Rodrigo Sierra,[136] Milton Tirado,[136] Maria Natalia Umaña,[137] Geertje van der Heijden,[138] Emilio Vilanova Torre,[134,139] Manuel Augusto Ahuite Reategui,[140] Cláudia Baider,[141,82] Henrik Balslev,[142] Sasha Cárdenas,[51] Luisa Fernanda Casas,[51] María José Endara,[143] William Farfan-Rios,[58,22] Cid Ferreira,[4†] Reynaldo Linares-Palomino,[61] Casimiro Mendoza,[144,145] Italo Mesones,[88] Germaine Alexander Parada,[52] Armando Torres-Lezama,[134] Ligia Estela Urrego Giraldo,[146] Daniel Villarroel,[52,147] Roderick Zagt,[148] Miguel N. Alexiades,[149] Edmar Almeida de Oliveira,[35] Karina Garcia-Cabrera,[58] Lionel Hernandez,[40] Walter Palacios Cuenca,[150] Susamar Pansini,[26] Daniela Pauletto,[151] Freddy Ramirez Arevalo,[152] Adeilza Felipe Sampaio,[26] Elvis H. Valderrama Sandoval,[153,152] Luis Valenzuela Gamarra,[31] Aurora Levesley,[13] Georgia Pickavance,[13] Karina Melgaço,[13]

*Author for correspondence hans.tersteege@naturalis.nl; †deceased

Contents

Supplementary Figs S1 – S31	4 – 33
Supplementary Results of species richness in a sample of 500 individuals	12
Supplementary Table S1	34
Supplementary References	35

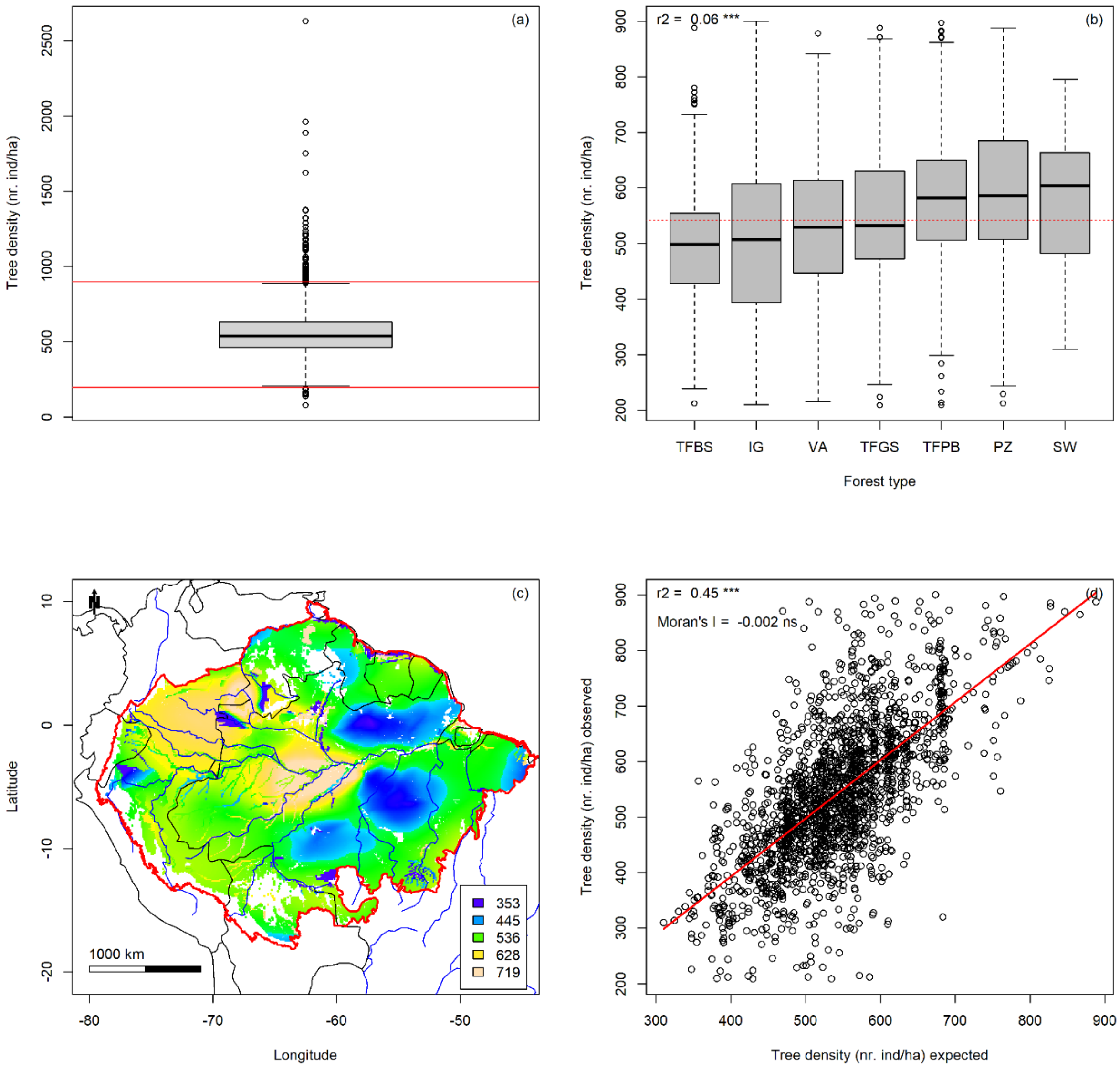

Supplementary Fig. 1. Tree-density (number of individuals/ha) in Amazonia. A) Boxplot of tree-density in 2,046 ATDN plots. Plots outside the 95% confidence interval were not used for spatial interpolation. **B)** Tree-density by major forest type. Red lines, mean ± 2 sd. **C)** Map of tree-density across Amazonia. Legend truncated at mean ± 2 standard deviations of the mean (see A). **D)** Observed values of tree-density vs modelled values of tree-density on the 1,956 plots used for mapping. The significance or Moran's I was tested with the function Moran.I() of ape [1]. Red polygon: Amazonian Biome limit [2]. Maps created with a custom R [3] script. Base map source (country.shp, rivers.shp): ESRI (http://www.esri.com/data/basemaps, © Esri, DeLorme Publishing Company).

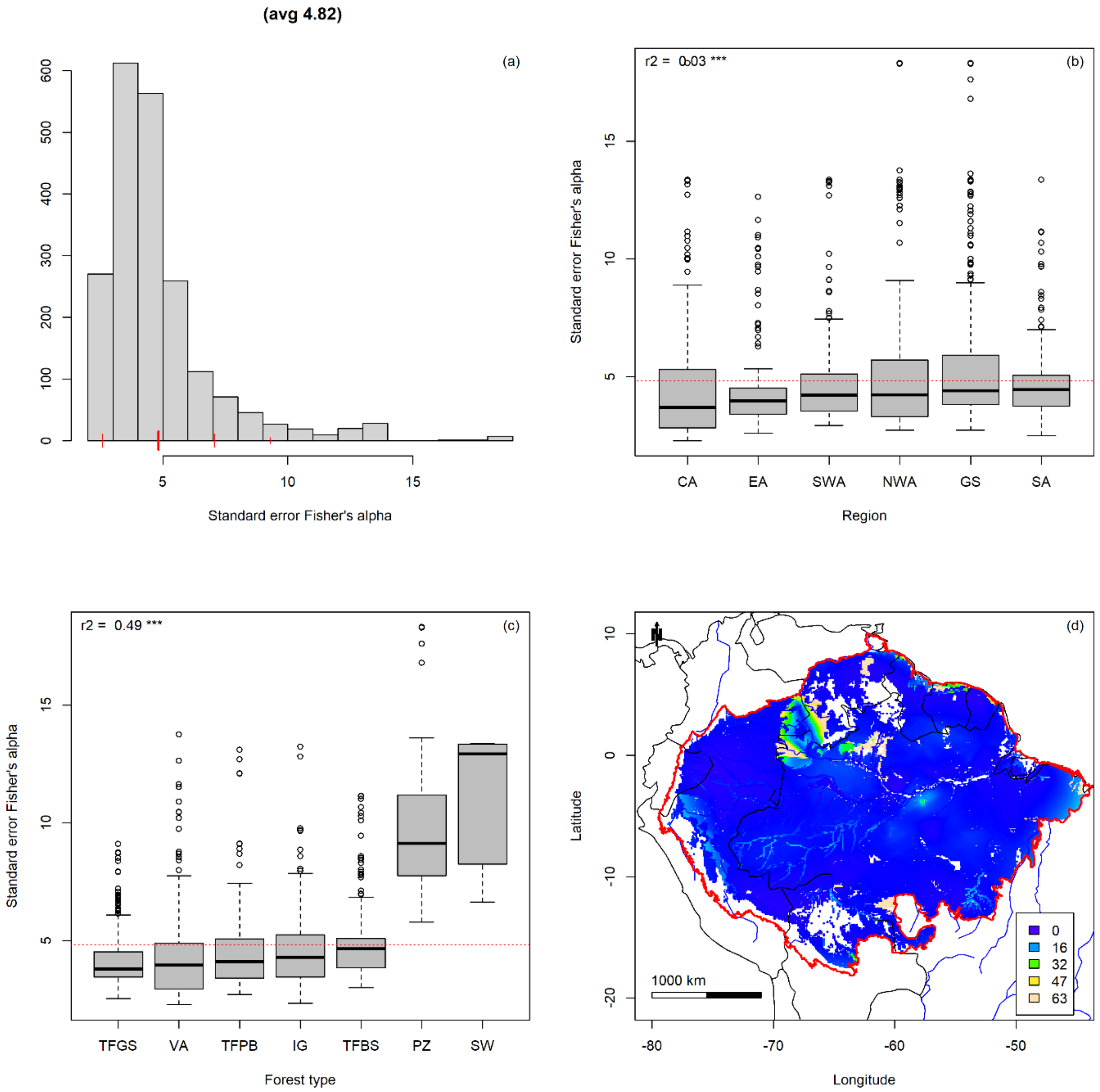

Supplementary Fig. 2. Standard error of loess interpolation of Fisher's alpha (Figure 1, main text). A) Histogram, showing the standard error of the loess predictions for tree alpha-diversity (Fisher's alpha) by plot. **B)** Standard error of the loess predictions for tree alpha-diversity by region. **C**) Standard error of the loess predictions for tree alpha-diversity by forest type. **D**) Map of the standard error of the loess predictions for tree alpha-diversity, showing high error in the white sand regions of Upper Rio Negro and Guianas and swamp forests of the latter. Red polygon: Amazonian Biome limit [2]. Maps created with a custom R [3] script. Base map source (country.shp, rivers.shp): ESRI (http://www.esri.com/data/basemaps, © Esri, DeLorme Publishing Company).

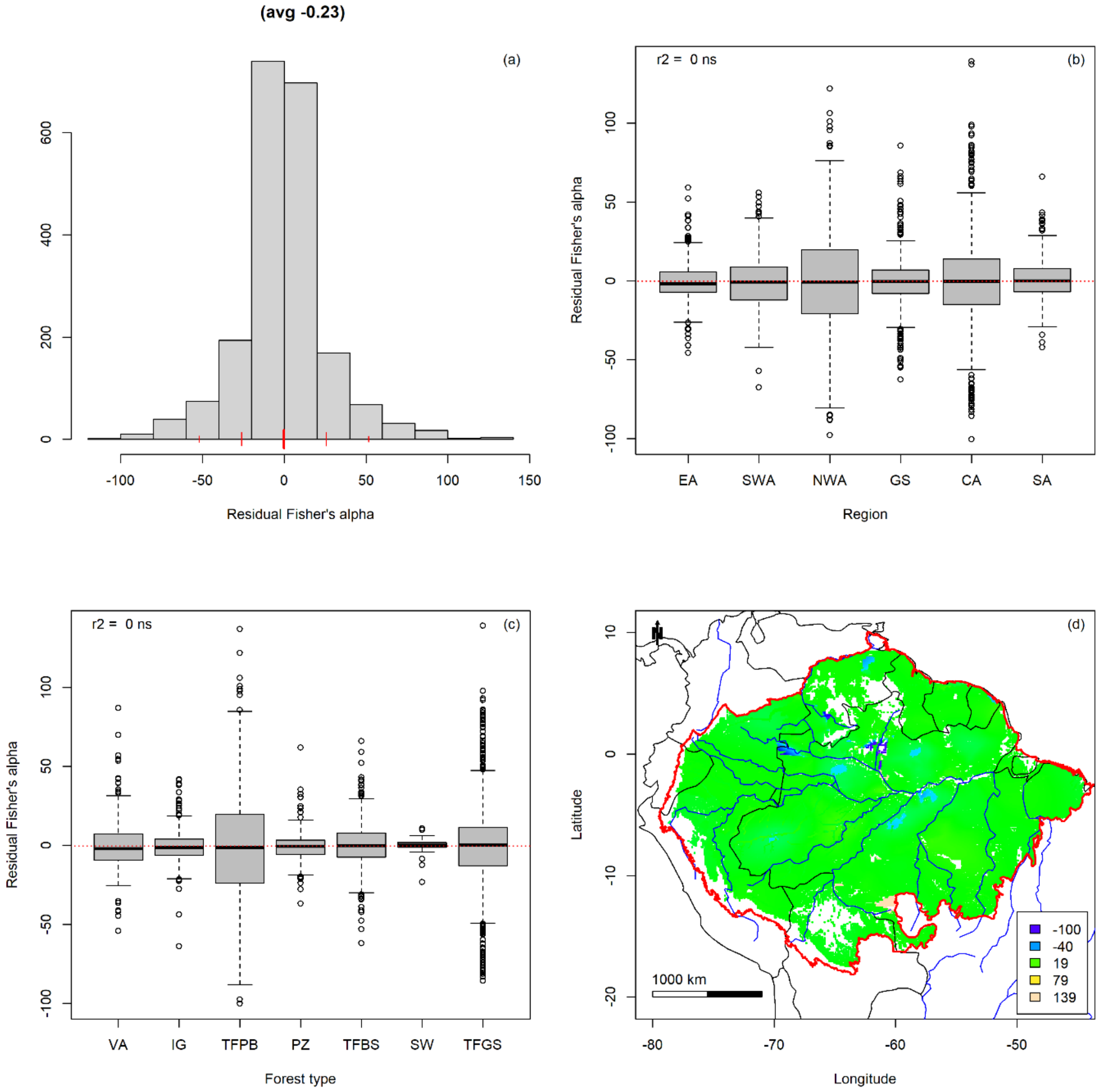

Supplementary Fig. 3. Residuals from loess interpolation of Fisher's alpha (Figure 1, main text). A) Histogram, showing 'normally' distributed residuals with mean close to zero. **B)** Residuals by region. Median of residuals of all regions close to zero. **C**) Residuals by forest type. Median of residuals of all forest types, except swamp forest close to zero. **D**) Map of residuals, showing no or weak spatial structure. Red polygon: Amazonian Biome limit [2]. Maps created with a custom R [3] script. Base map source (country.shp, rivers.shp): ESRI (http://www.esri.com/data/basemaps, © Esri, DeLorme Publishing Company).

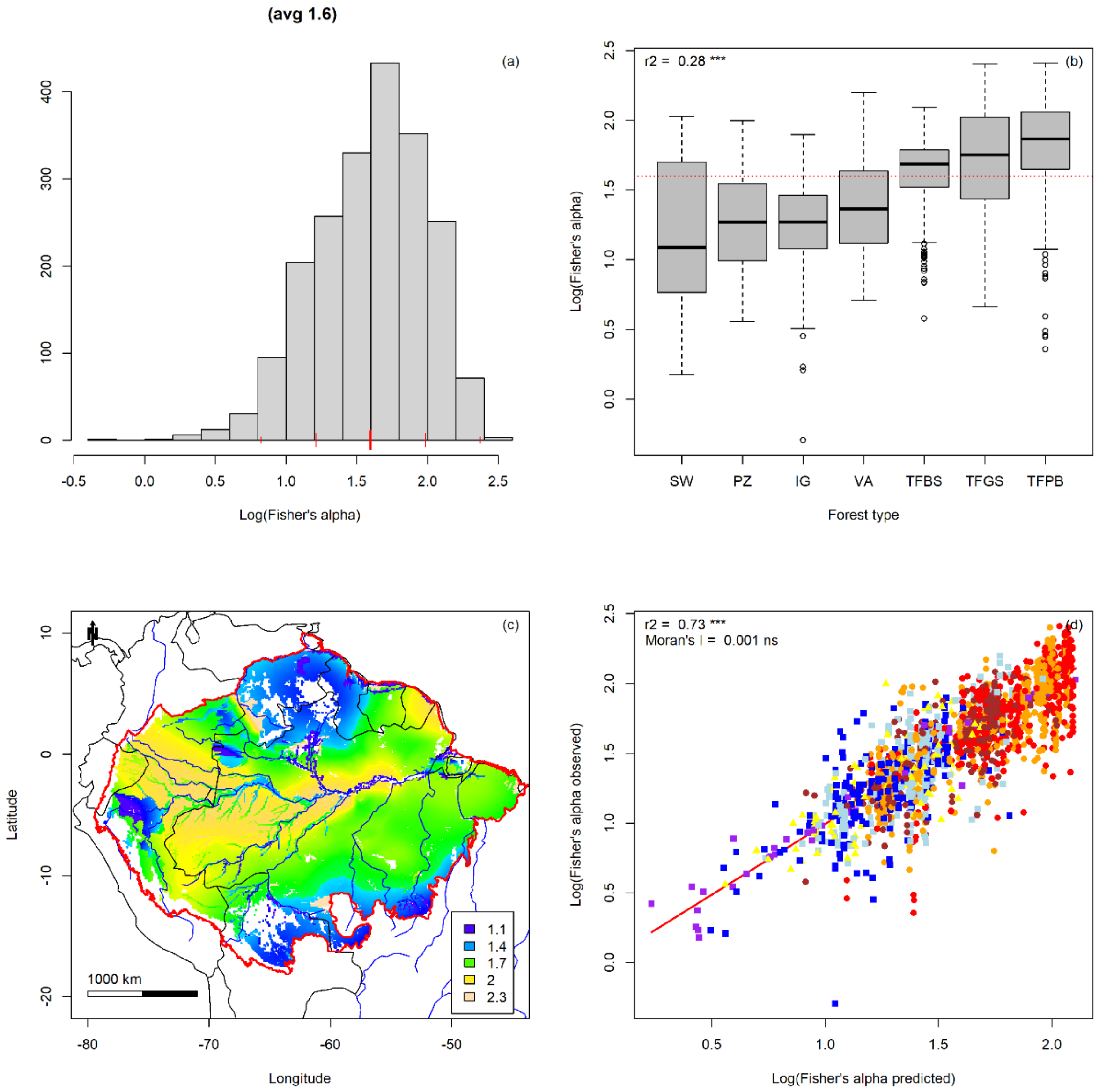

Supplementary Fig. 4. Tree alpha-diversity (Fisher's alpha) in Amazonia – logarithmic version. A) Histogram of ^{10}log(Fisher's alpha) in 2,046 ATDN plots. Red lines, mean and mean ± 2 sd. **B)** Fisher's alpha by major forest type. **C)** Map of Fisher's alpha across Amazonia. Legend truncated at 0 and mean + 2 standard deviation of the mean. Amazonian Biome limit - red [2]. **D**) Observed values of tree diversity vs modelled values of tree diversity on the 2,046 plots used for mapping. The significance or Moran's I was tested with the function Moran.I() of ape [1]. Marker colors: Red: Terra Firme Pebas Formation; Brown: Terra Firme Brazilian Shield; Orange: Terra Firme Guyana Shield; Yellow: White sand forest; Light blue: Varzea; Dark blue: Igapo; Purple: Swamp. Map created with custom R [3] script. Base map source (country.shp, rivers.shp): ESRI (http://www.esri.com/data/basemaps, © Esri, DeLorme Publishing Company).

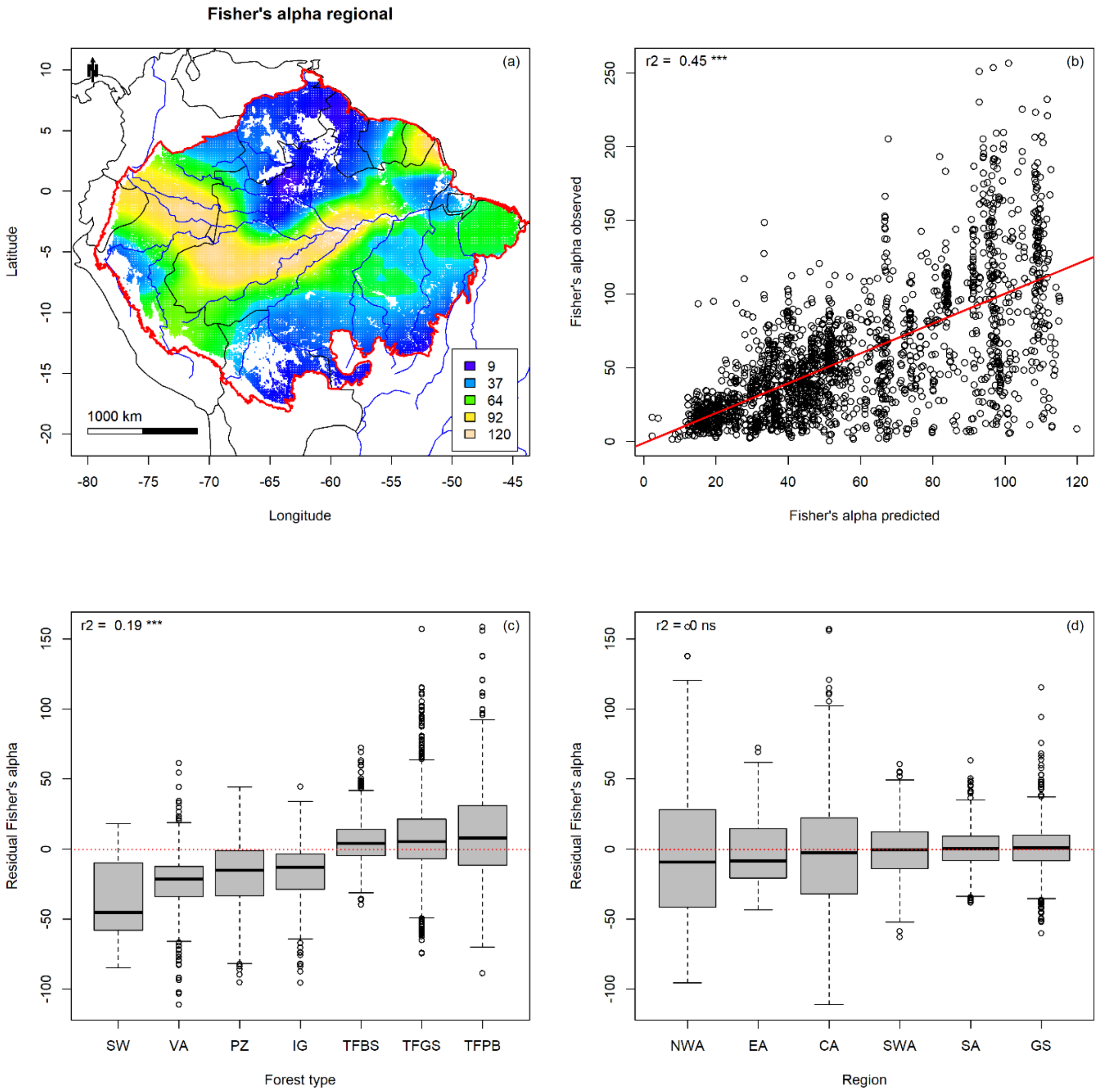

Supplementary Fig. 5. Mapping Fisher's alpha unstratified. Map of Fisher's alpha not using forest types but methods as refs [4,5] **A**) Map of Fisher's alpha at 0.1 degree. **B)** Predicted vs. observed Fisher's alpha by plot. **C**) Residuals by forest type. Median of residuals of TF close to zero. **D**) Residuals by region, showing no or weak spatial structure. Red polygon: Amazonian Biome limit [2]. Maps created with a custom R [3] script. Base map source (country.shp, rivers.shp): ESRI (http://www.esri.com/data/basemaps, © Esri, DeLorme Publishing Company).

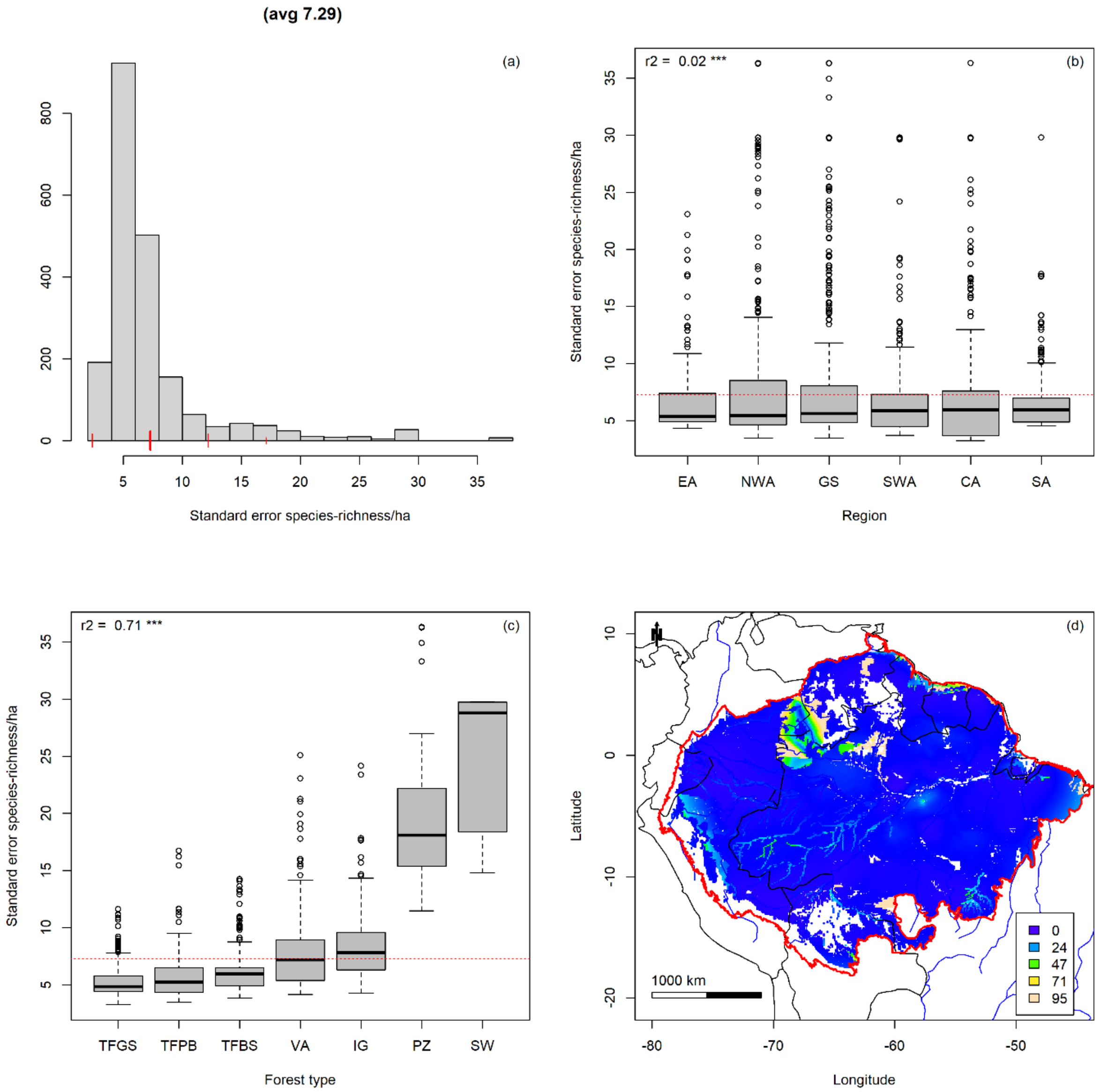

Supplementary Fig. 6. Standard error of loess interpolation of species-richness per ha (Figure 2, main text). A) Histogram, showing the standard error of the loess predictions for tree species-richness by ha. **B)** Standard error of the loess predictions for tree species-richness by region. **C**) Standard error of the loess predictions for tree species-richness by forest type. **D**) Map of the standard error of the loess predictions for tree species-richness, showing high error in the white sand regions of Upper Rio Negro and Guianas and swamp forests of the latter. Red polygon: Amazonian Biome limit [2]. Maps created with a custom R [3] script. Base map source (country.shp, rivers.shp): ESRI (http://www.esri.com/data/basemaps, © Esri, DeLorme Publishing Company).

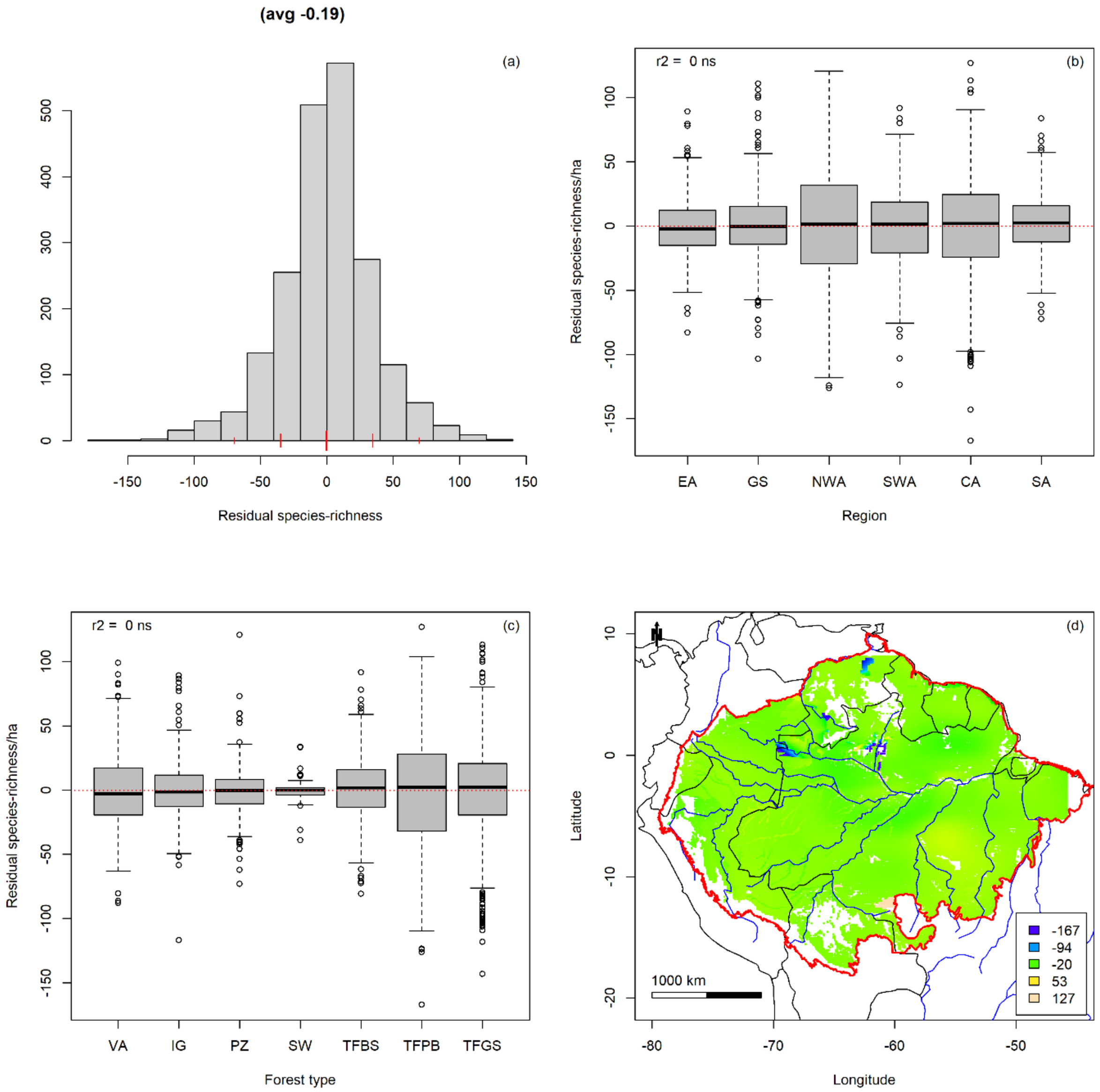

Supplementary Fig. 7. Residuals from loess interpolation of species-richness per ha (Figure 2, main text). A) Histogram, showing 'normally' distributed residuals with mean close to zero. **B)** Residuals by region. Median of residuals of all regions close to zero. **C**) Residuals by forest type. Median of residuals of all forest types, except swamp forest, close to zero. **D**) Map of residuals, showing no or weak spatial structure. Red polygon: Amazonian Biome limit [2]. Maps created with a custom R [3] script. Base map source (country.shp, rivers.shp): ESRI (http://www.esri.com/data/basemaps, © Esri, DeLorme Publishing Company).

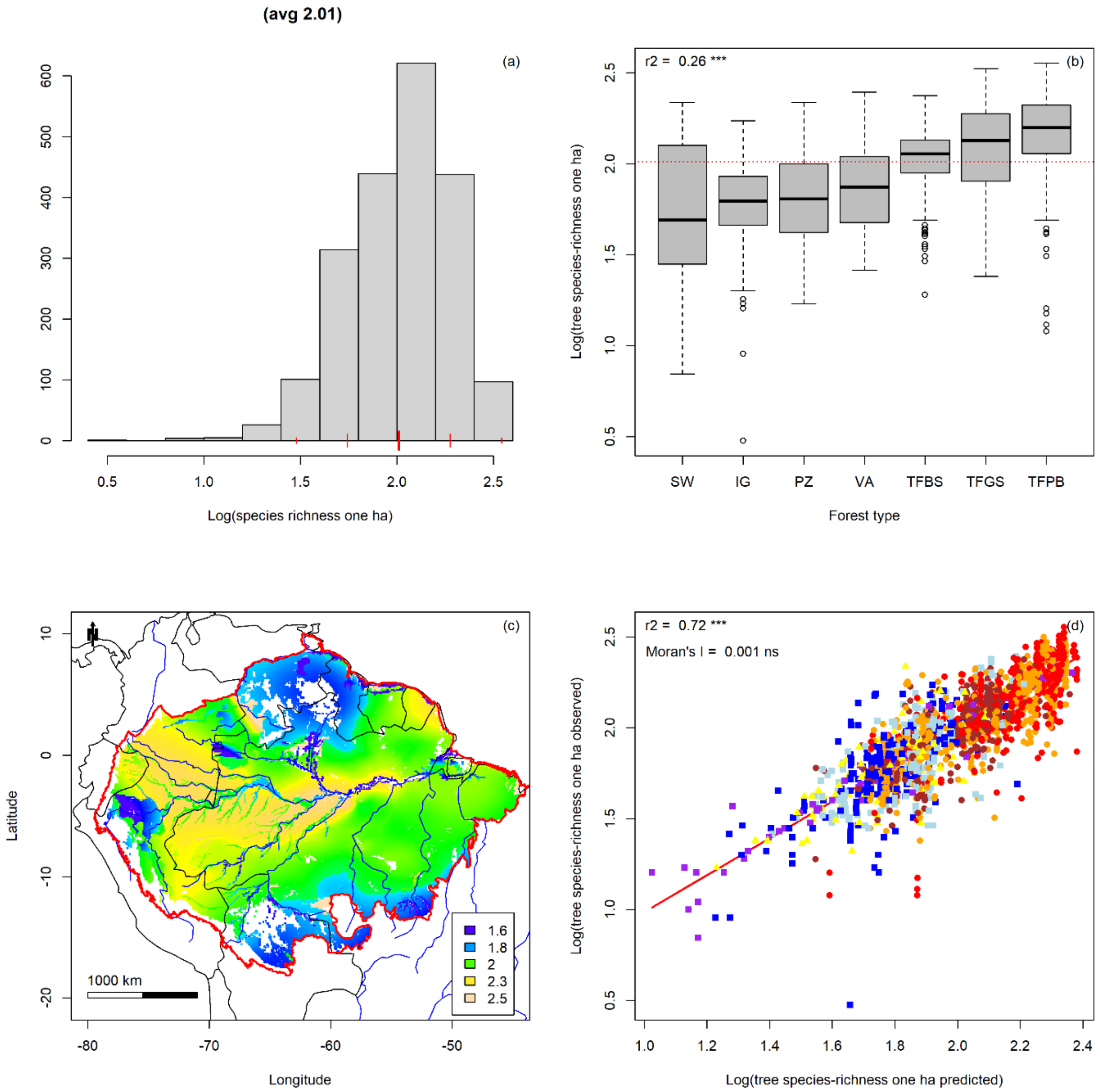

Supplementary Fig. 8. Tree species-richness in Amazonia – logarithmic version. A) Histogram of ^{10}log(tree species-richness) in 2,046 ATDN plots. **B)** ^{10}log(tree species-richness) by major forest type. **C)** Map of tree ^{10}log(tree species-richness) across Amazonia. Legend truncated at mean ± 2 standard deviations of the mean. Amazonian Biome limit - red [2]. **D**) Observed values of ^{10}log(tree species-richness) vs modelled values of ^{10}log(tree species-richness) on the 2,046 plots used for mapping. The significance or Moran's I was tested with the function Moran.I() of ape [1]. Marker colors: Red: Terra Firme Pebas Formation; Brown: Terra Firme Brazilian Shield; Orange: Terra Firme Guyana Shield; Yellow: White sand forest; Light blue: Varzea; Dark blue: Igapo; Purple: Swamp. Map created with custom R [3] script. Base map source (country.shp, rivers.shp): ESRI (http://www.esri.com/data/basemaps, © Esri, DeLorme Publishing Company).

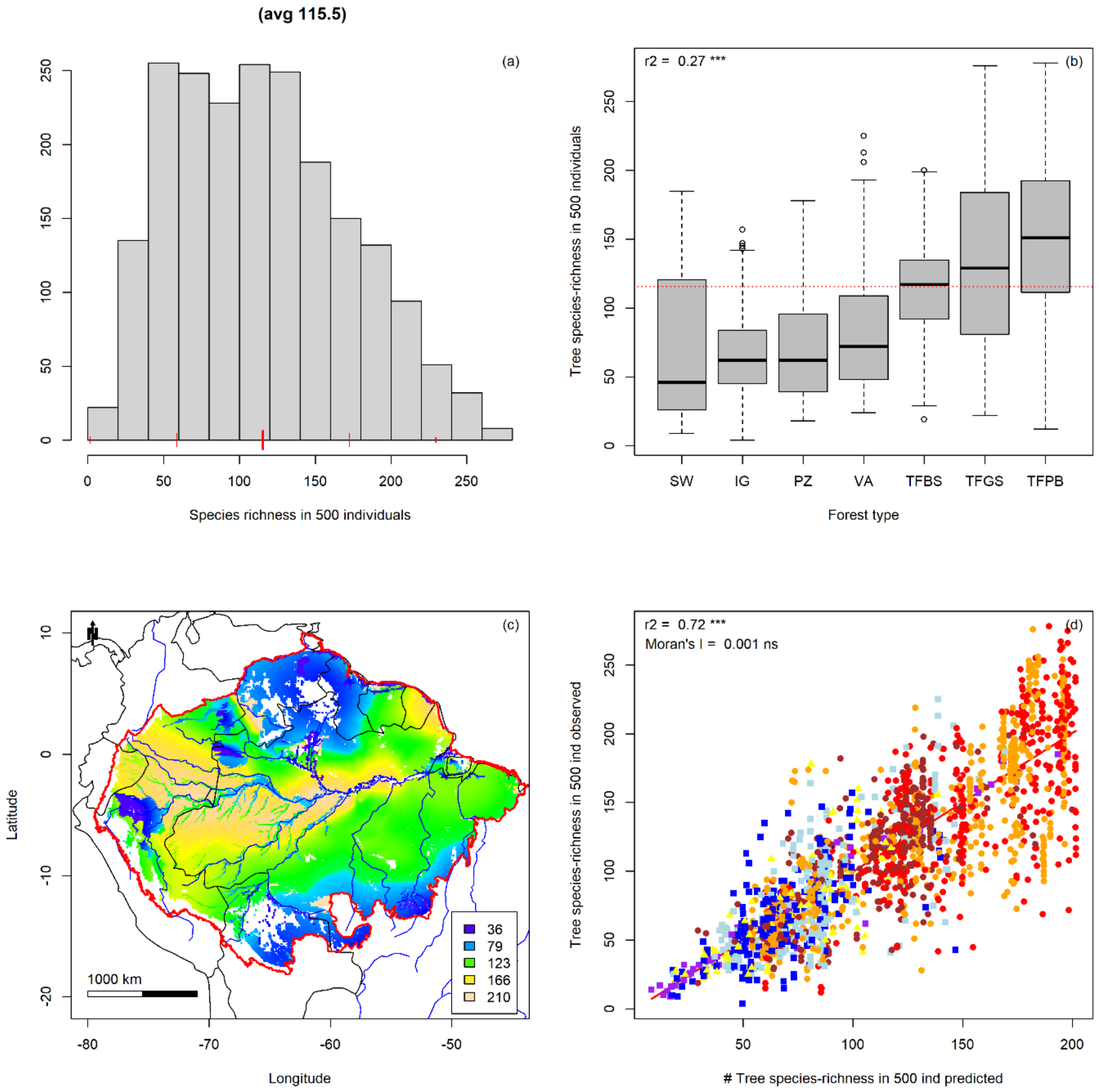

Supplementary Fig. 9. Tree species-richness (species/500 individuals) in Amazonia. A) Histogram of tree species-richness in 2,046 ATDN plots. **B**) Tree species-richness by major forest type. **C)** Map of tree species-richness across Amazonia. Legend truncated at mean ± 2 standard deviations of the mean. **D**) Observed values of tree species-richness vs modelled values of tree species-richness on the 2,046 plots used for mapping. The significance or Moran's I was tested with the function Moran.I() of ape [1]. Red polygon: Amazonian Biome limit [2]. Maps created with a custom R [3] script. Base map source (country.shp, rivers.shp): ESRI (http://www.esri.com/data/basemaps, © Esri, DeLorme Publishing Company).

Tree species-richness, here defined as the number of species per 500 individuals, ranged from 4 to 278, with an average of 116 species per 500 individuals. The data was less skewed than that of diversity based on tree species-richness per ha (Supplementary Fig. 9A). Forest type explained a significant amount of variation in species-richness ($R^2 = 27\%$, $p < 0.001$) (Supplementary Fig. 9B). Highest species-richness was found in the terra-firme forests of the Pebas region (western Amazonia) and French Guiana (Supplementary Fig. 9C). Lowest species-richness was again found on the sandy soils of Guyana, Upper Rio Negro, southern Amazonia, and on floodplains along the rivers and swamps. The combined spatial model explained 70% of the variation in tree species-richness across Amazonia (Supplementary Fig. 9D) and its residuals showed no significant spatial autocorrelation (Moran's I = 0.001 ns).

The standard error for tree species-richness was mostly low (Supplementary Fig. 10A) and rather constant across regions (Supplementary Fig. 10B) but higher for white sand forest and swamp forests (Supplementary Fig. 10C), resulting in higher standard errors in the white sand areas of the Upper Rio Negro and Guianas (Supplementary Fig. 10C).

Residuals of the combined spatial model had a mean of close to zero and did not differ much between forest type and region and very little spatial pattern (Supplementary Fig. 11).

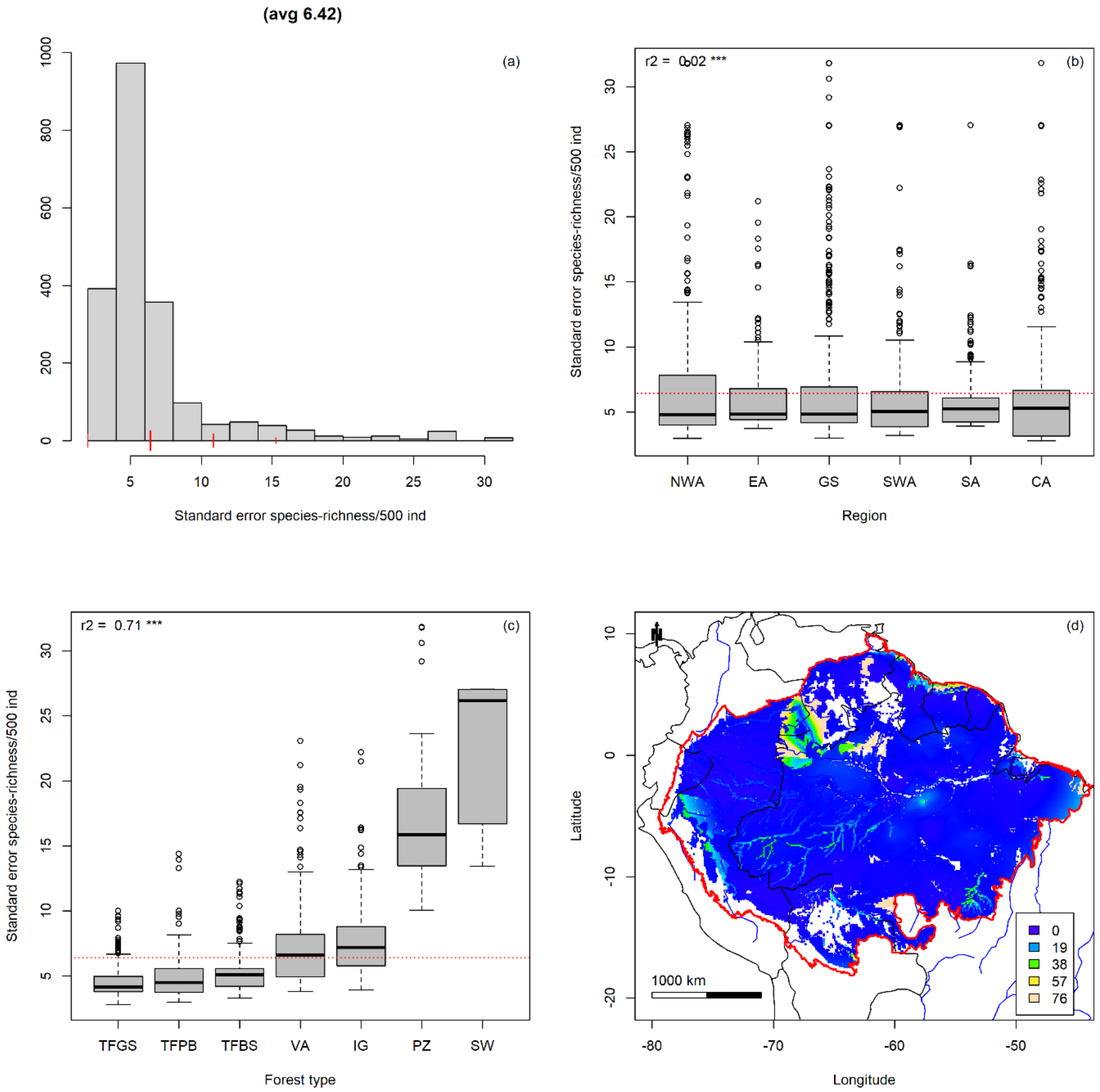

Supplementary Fig. 10. Standard error of loess interpolation of species-richness per 500 individuals. A) Histogram, showing the standard error of the loess predictions for tree species-richness by plot. **B)** Standard error of the loess predictions for tree species-richness by region. **C**) Standard error of the loess predictions for tree species-richness by forest type. **D**) Map of the standard error of the loess predictions for tree species-richness, showing high error in the white sand regions of Upper Rio Negro and Guianas and swamp forests of the latter. Red polygon: Amazonian Biome limit [2]. Maps created with a custom R [3] script. Base map source (country.shp, rivers.shp): ESRI (http://www.esri.com/data/basemaps, © Esri, DeLorme Publishing Company).

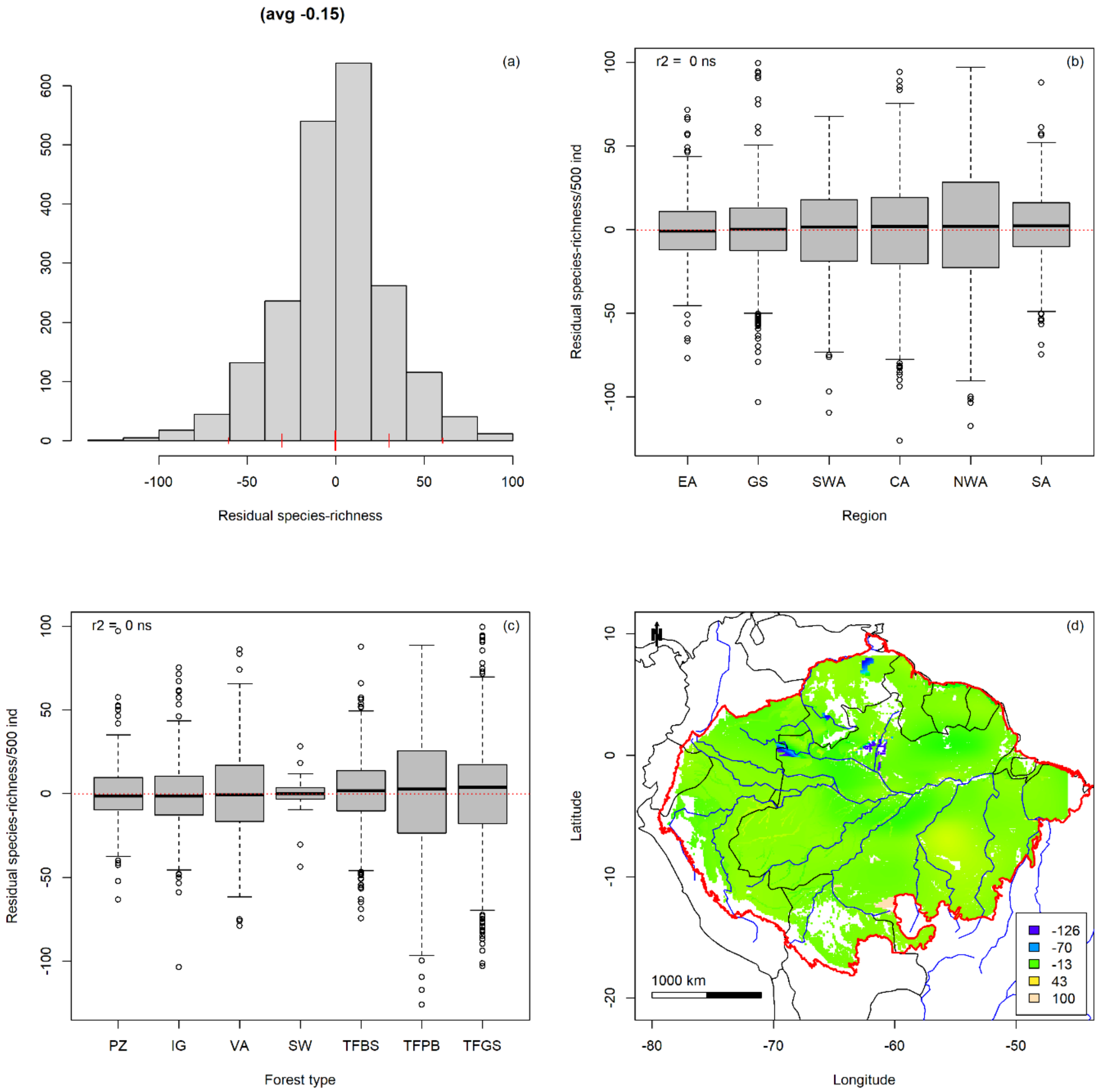

Supplementary Fig. 11. Residuals from loess interpolation of species-richness per 500 ind. A) Histogram, showing 'normally' distributed residuals with mean close to zero. **B)** Residuals by region. Median of residuals of all regions close to zero. **C**) Residuals by forest type. Median of residuals of all forest types, except swamp forest, close to zero. **D**) Map of residuals, showing no or weak spatial structure. Red polygon: Amazonian Biome limit [2]. Maps created with a custom R [3] script. Base map source (country.shp, rivers.shp): ESRI (http://www.esri.com/data/basemaps, © Esri, DeLorme Publishing Company).

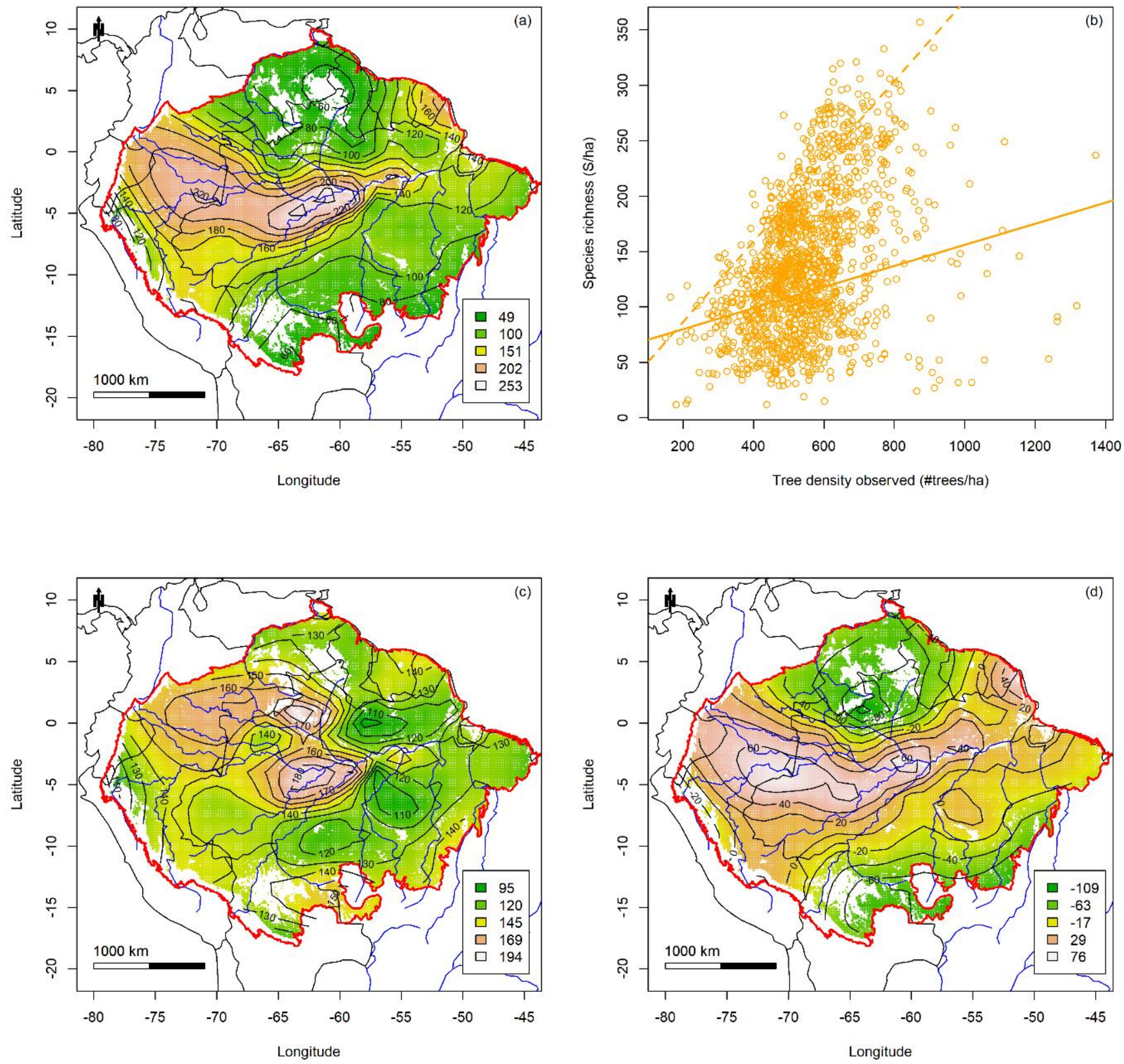

Supplementary Fig. 12. **The effect of plot tree-density (# individuals / ha) on tree species-richness**. **A**) Tree species-richness observed. **B**) Tree species-richness vs. plot tree-density. Thick line – quantile regression with tau = 0.5 (equivalent to least absolute deviation regression; least squares regression: R^2 = 16.5%); thin dashed line – quantile regression with tau = 0.9, defining the upper limit of the data. **C**) Tree species-richness as predicted by plot tree-density. **D**) Residuals of tree species-richness predicted by plot tree-density (**A** – **C**). Red polygon: Amazonian Biome limit [2]. Maps created with a custom R [3] script. Base map source (country.shp, rivers.shp): ESRI (http://www.esri.com/data/basemaps, © Esri, DeLorme Publishing Company).

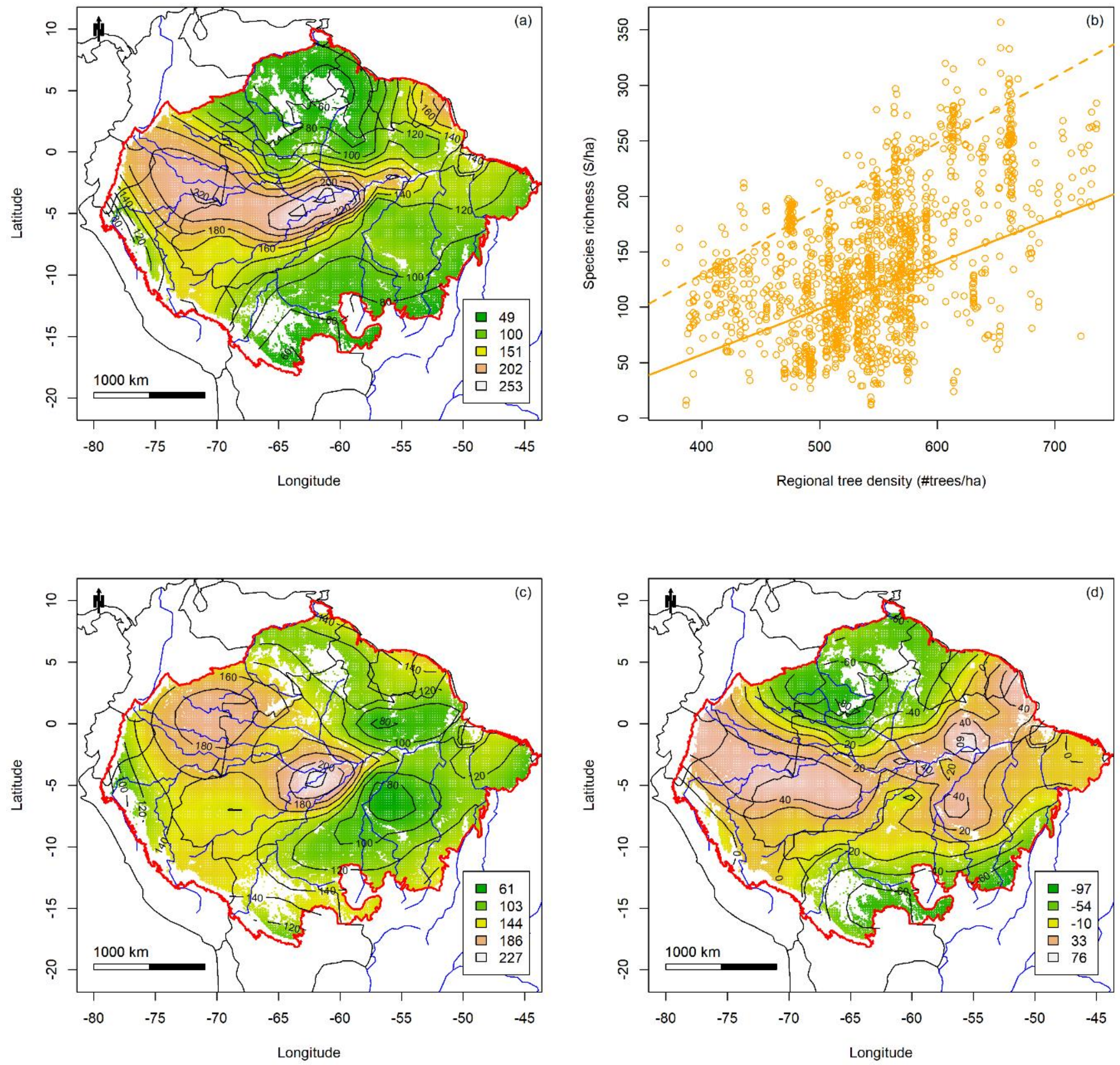

Supplementary Fig. 13. **The effect of regional tree-density (# individuals / ha) on tree species-richness. A)** Tree species-richness observed. **B**) Tree species-richness vs. regional tree-density. Thick line – quantile regression with tau = 0.5 (equivalent to least absolute deviation regression; least squares regression: R^2 = 24%); thin dashed line – quantile regression with tau = 0.9, defining the upper limit of the data. **C)** Tree species-richness as predicted by regional tree-density. **D)** Residuals of tree species-richness predicted by regional tree-density (**A** – **C**). Red polygon: Amazonian Biome limit [2]. Maps created with a custom R [3] script. Base map source (country.shp, rivers.shp): ESRI (http://www.esri.com/data/basemaps, © Esri, DeLorme Publishing Company).

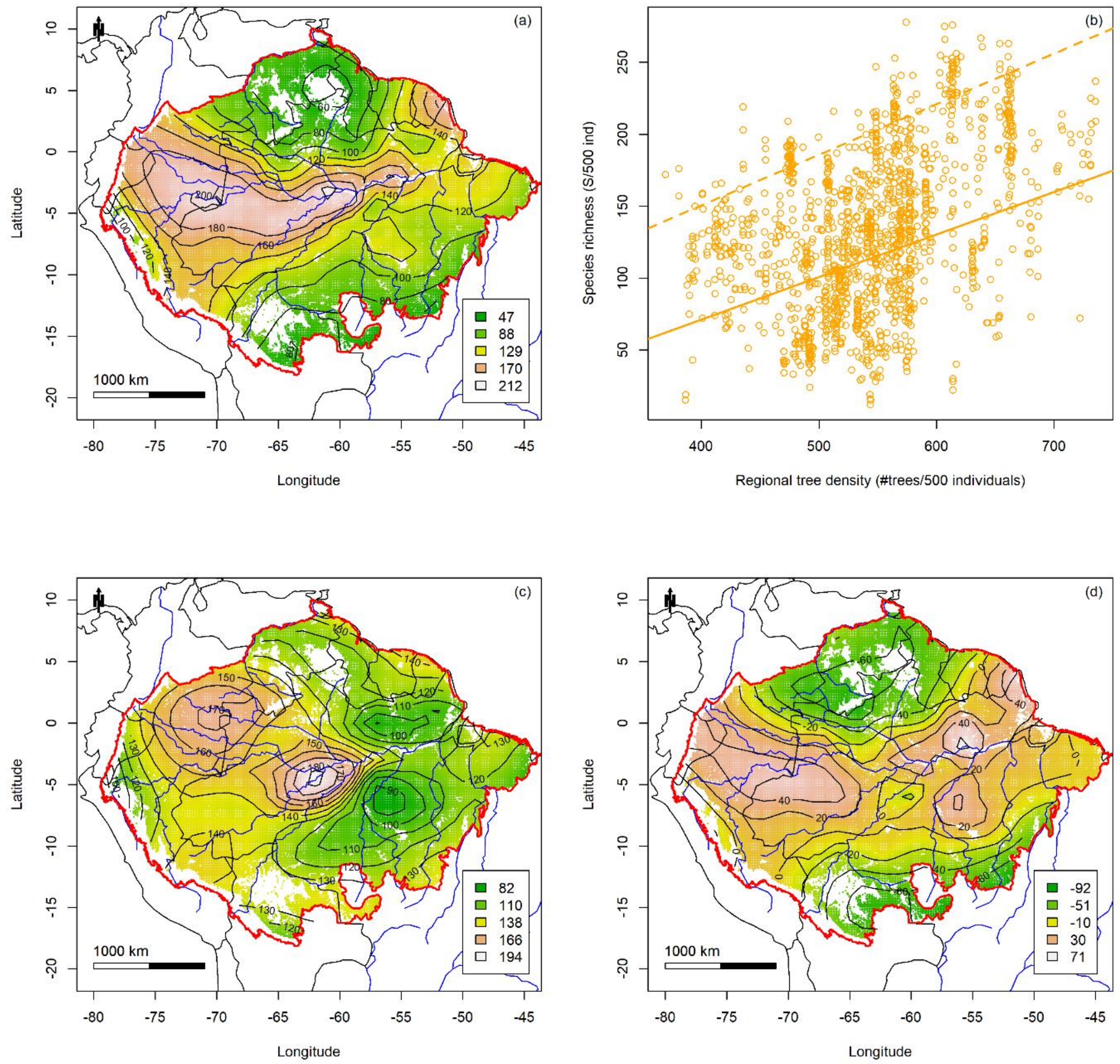

Supplementary Fig. 14. **The effect of regional tree-density (# individuals / ha) on tree species-richness per 500 ind. A**) Tree species-richness / 500ind. observed. **B**) Tree species-richness vs. regional tree-density (equivalent to least absolute deviation regression; least squares regression: R^2 = 14%). Thick line – quantile regression with tau = 0.5 (equivalent to least absolute deviation regression); thin dashed line – quantile regression with tau = 0.9, defining the upper limit of the data. **C**) Tree species-richness as predicted by regional tree-density. **D**) Residuals of tree species-richness predicted by regional tree-density (**A** – **C**). Red polygon: Amazonian Biome limit [2]. Maps created with a custom R [3] script. Base map source (country.shp, rivers.shp): ESRI (http://www.esri.com/data/basemaps, © Esri, DeLorme Publishing Company).

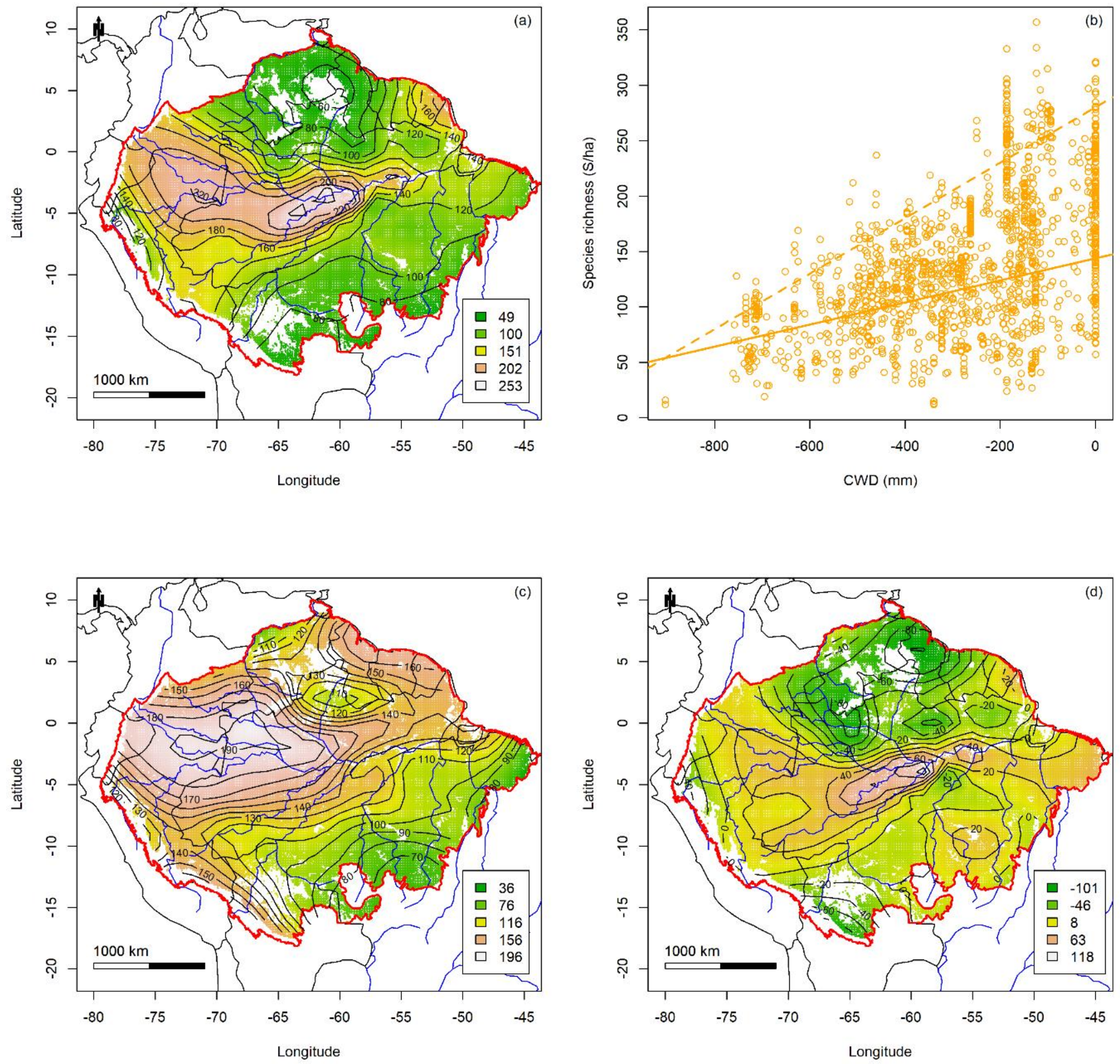

Supplementary Fig. 15. **The effect of cumulative water deficit (mm) on tree species-richness. A**) Tree species-richness observed. **B**) Tree species-richness vs. cumulative water deficit (CWD) (mm). Thick line – quantile regression with tau = 0.5 (equivalent to least absolute deviation regression; least squares regression: R^2 = 27%); thin dashed line – quantile regression with tau = 0.9, defining the upper limit of the data. **C**) Tree species-richness as predicted by cumulative water deficit. **D**) Interpolated residuals of the quantile regression (tau = 0.5). Red polygon: Amazonian Biome limit [2]. Maps created with a custom R [3] script. Base map source (country.shp, rivers.shp): ESRI (http://www.esri.com/data/basemaps, © Esri, DeLorme Publishing Company).

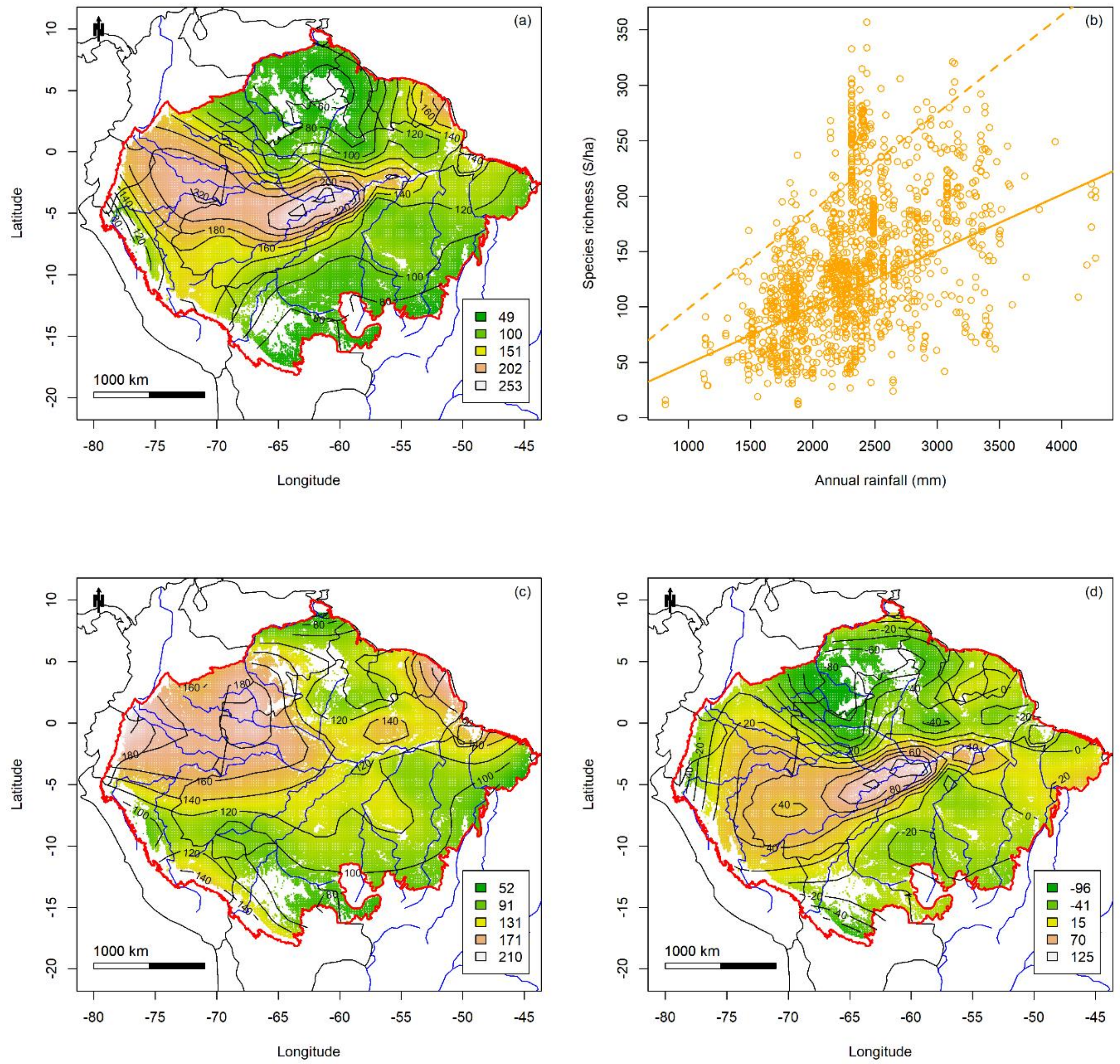

Supplementary Fig. 16. **Species-richness/ha as a function of Annual rainfall**. **A**) Tree species-richness observed. **B**) Tree species-richness vs. annuals rainfall (mm/year). Thick line – quantile regression with tau = 0.5 (equivalent to least absolute deviation regression); thin dashed line – quantile regression with tau = 0.9, defining the upper limit of the data. **C**) Tree species-richness as predicted by annual rainfall. **D)** Interpolated residuals of the quantile regression (tau = 0.5). Red polygon: Amazonian Biome limit [2]. Maps created with a custom R [3] script. Base map source (country.shp, rivers.shp): ESRI (http://www.esri.com/data/basemaps, © Esri, DeLorme Publishing Company).

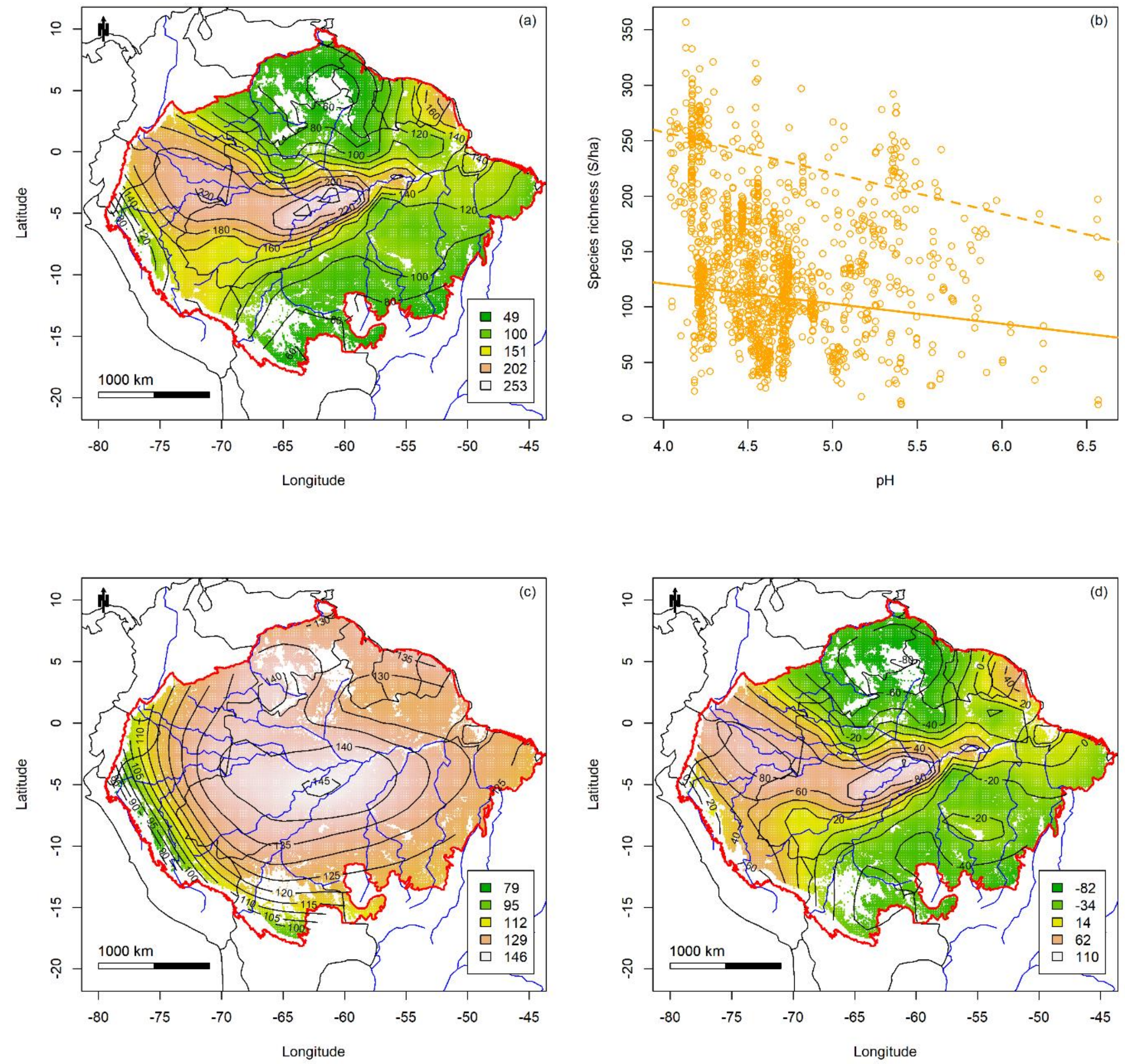

Supplementary Fig. 17. **Species-richness/ha soil and soil acidity (pH)**. **A**) Tree species-richness observed. **B**) Tree species-richness vs. pH. Thick line – quantile regression with tau = 0.5 (equivalent to least absolute deviation regression); thin dashed line – quantile regression with tau = 0.9, defining the upper limit of the data. **C**) Tree species-richness as predicted by pH. **D**) Interpolated residuals of the quantile regression (tau = 0.5). Red polygon: Amazonian Biome limit [2]. Maps created with a custom R [3] script. Base map source (country.shp, rivers.shp): ESRI (http://www.esri.com/data/basemaps, © Esri, DeLorme Publishing Company).

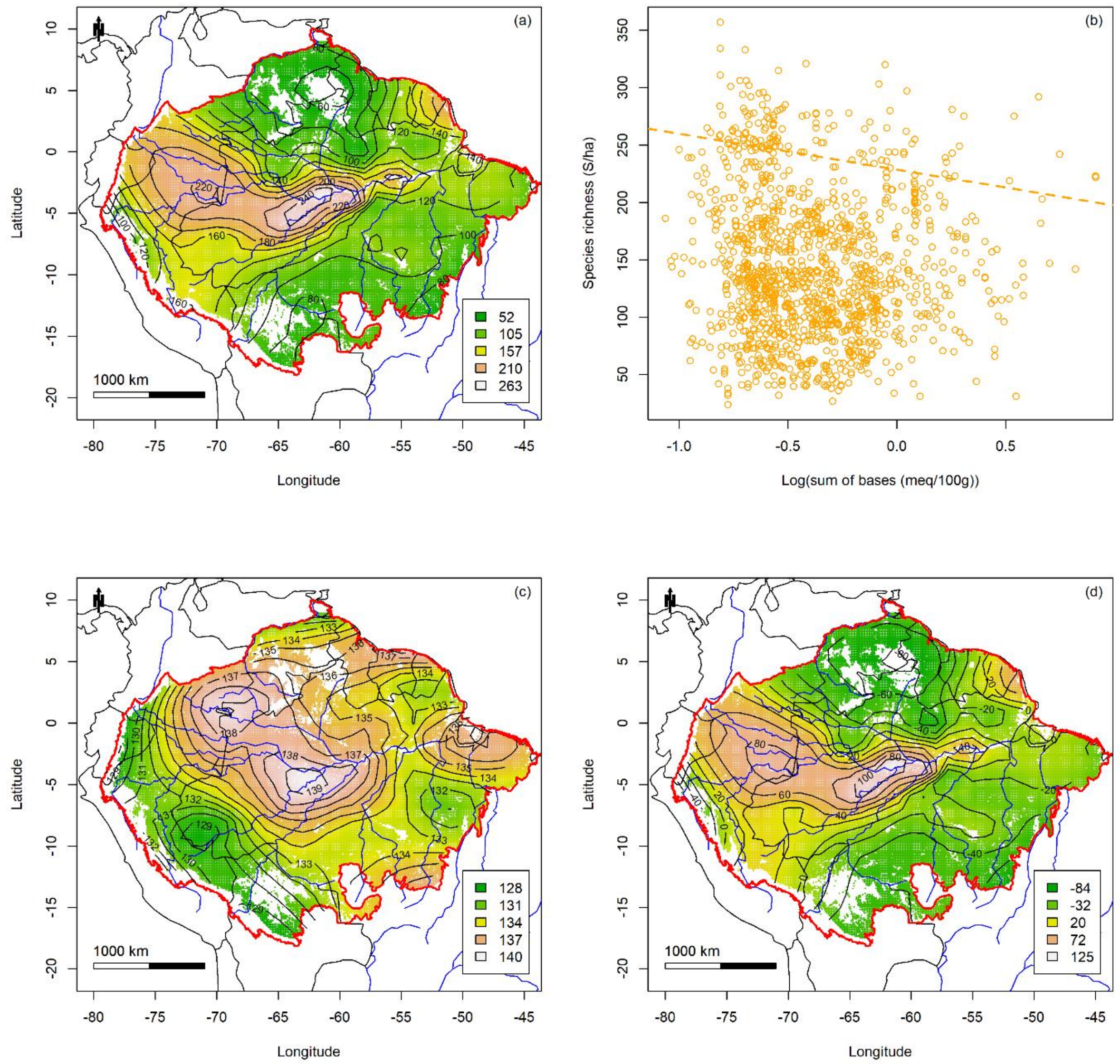

Supplementary Fig. 18. **Species-richness/ha as a function of soil fertility (log(sum of bases [meq/100g]))**. **A**) Tree species-richness observed. **B**) Tree species-richness vs. log(sum of bases (meq/100g)) [6]. Thick line – quantile regression with tau = 0.5 (equivalent to least absolute deviation regression); thin dashed line – quantile regression with tau = 0.9, defining the upper limit of the data. **C**) Tree species-richness as predicted by log(sum of bases). **D**) Interpolated residuals of the quantile regression (tau = 0.5). Red polygon: Amazonian Biome limit [2]. Maps created with a custom R [3] script. Base map source (country.shp, rivers.shp): ESRI (http://www.esri.com/data/basemaps, © Esri, DeLorme Publishing Company).

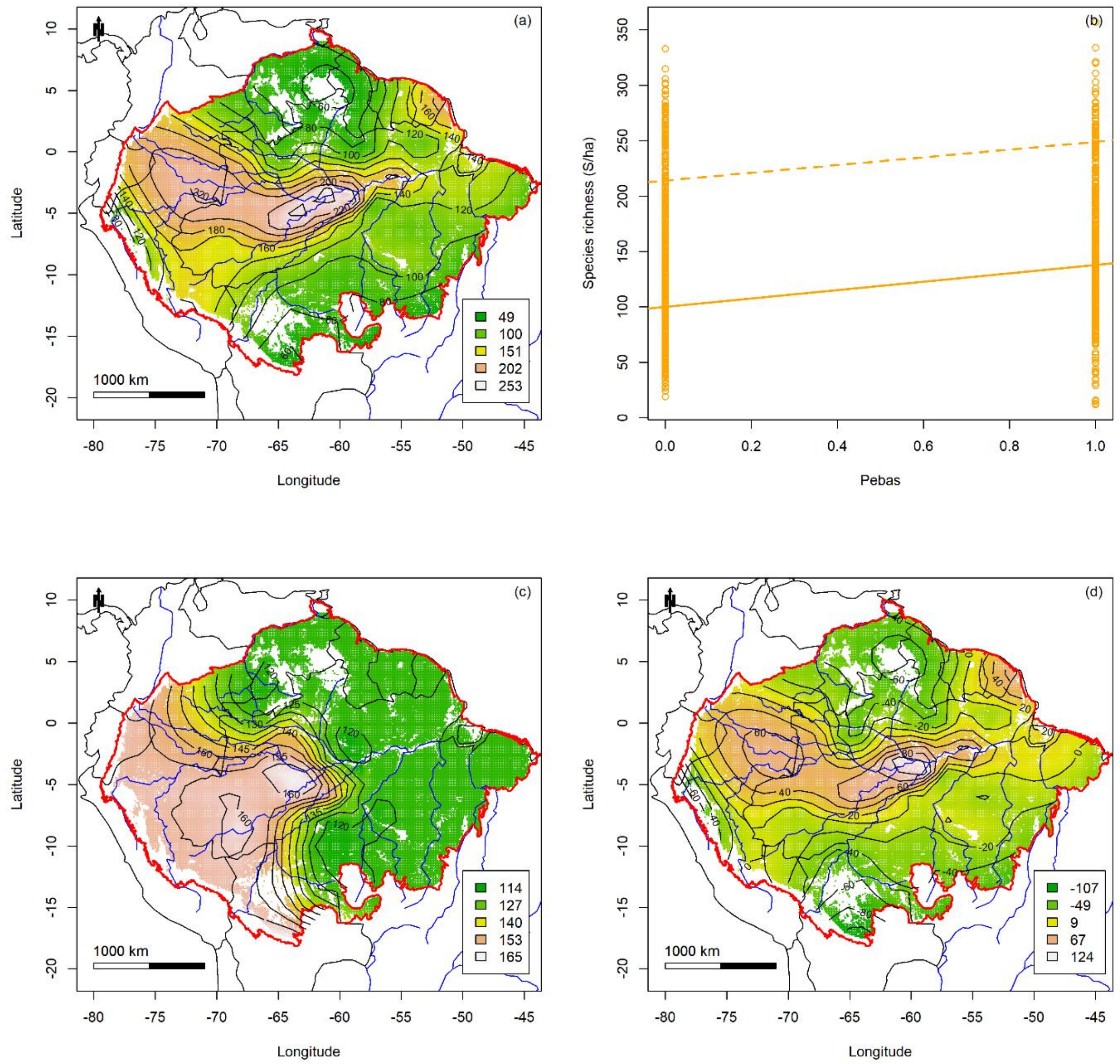

Supplementary Fig. 19. **Species-richness/ha soil fertility (location on Pebas)**. **A**) Tree species-richness observed. **B**) Tree species-richness vs. location on Pebas formation (1) or not (0). Thick line – quantile regression with tau = 0.5 (equivalent to least absolute deviation regression); thin dashed line – quantile regression with tau = 0.9, defining the upper limit of the data. **C**) Tree species-richness as predicted by location on Pebas (or not). **D**) Interpolated residuals of the quantile regression (tau = 0.5). Red polygon: Amazonian Biome limit [2]. Maps created with a custom R [3] script. Base map source (country.shp, rivers.shp): ESRI (http://www.esri.com/data/basemaps, © Esri, DeLorme Publishing Company).

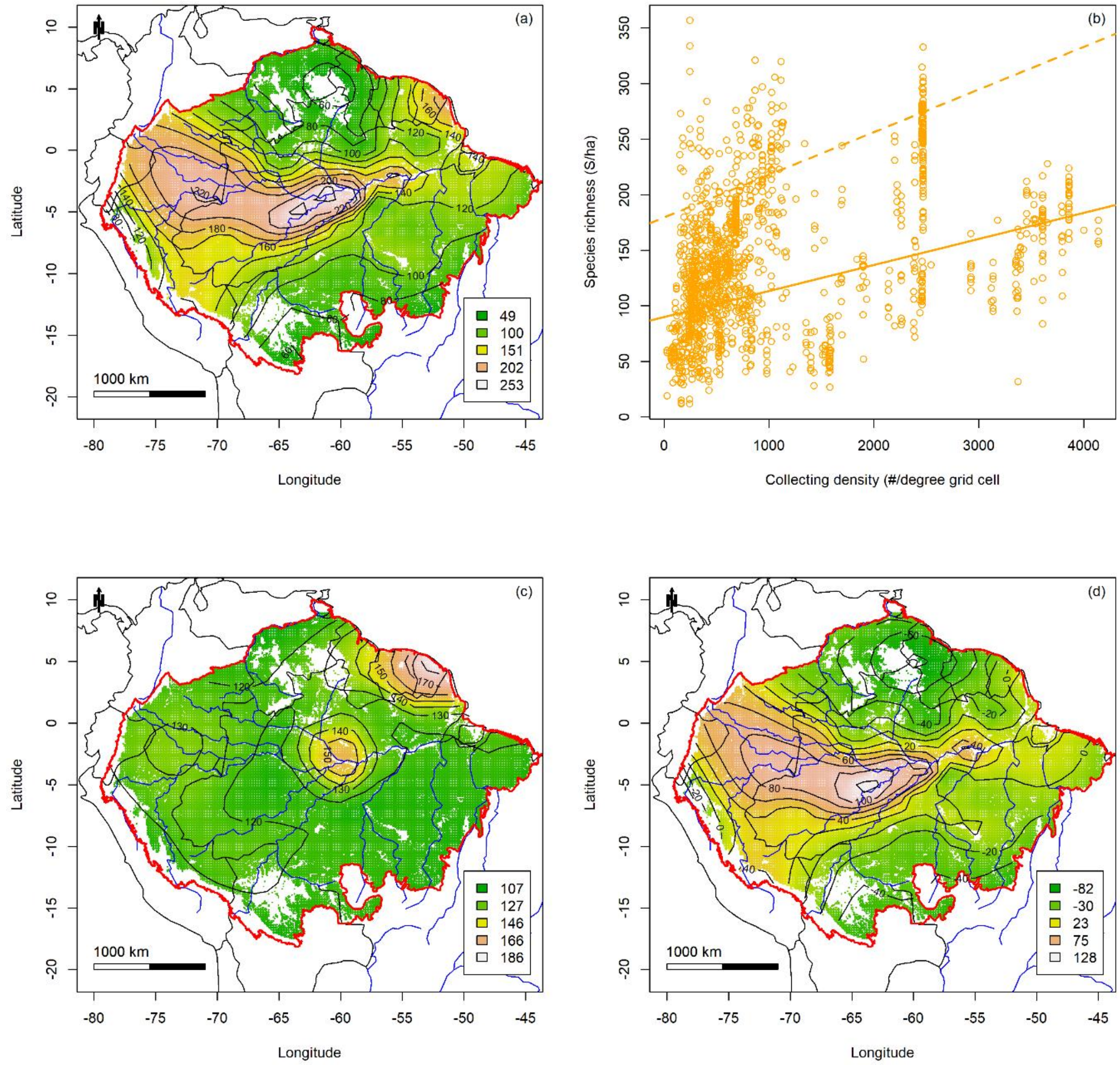

Supplementary Fig. 20. **The effect of collecting intensity (# collections) on tree species-richness**. **A**) Tree species-richness observed. **B**) Tree species-richness vs. collecting intensity (collections/1 degree grid cell) (mm). Thick line – quantile regression with tau = 0.5 (equivalent to least absolute deviation regression; least squares regression: R^2 = 13%); thin dashed line – quantile regression with tau = 0.9, defining the upper limit of the data. **C**) Tree species-richness as predicted by collecting intensity. **D**) Interpolated residuals of the quantile regression (tau = 0.5). Red polygon: Amazonian Biome limit [2]. Maps created with a custom R [3] script. Base map source (country.shp, rivers.shp): ESRI (http://www.esri.com/data/basemaps, © Esri, DeLorme Publishing Company).

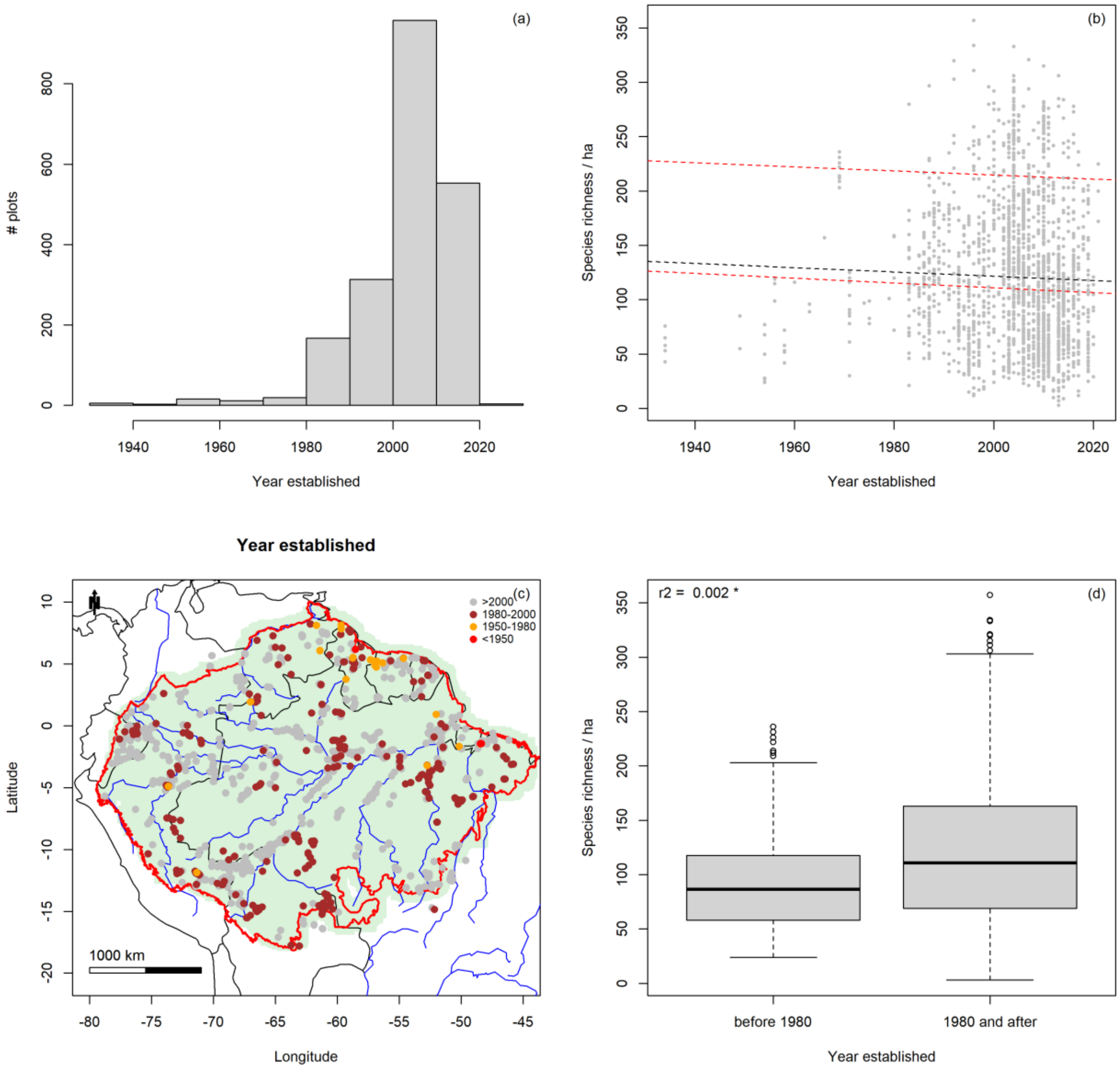

Supplementary Fig. 21. **The effect of year of establishment on tree species-richness. A**) Histogram of year established. **B)** Tree species-richness year established. **C)** Plots establishment started in eastern Amazonia **D)** Plots established after 1980 have higher species-richness/ha, the effect is very small but significant. Red polygon: Amazonian Biome limit [2]. Maps created with a custom R [3] script. Base map source (country.shp, rivers.shp): ESRI (http://www.esri.com/data/basemaps, © Esri, DeLorme Publishing Company).

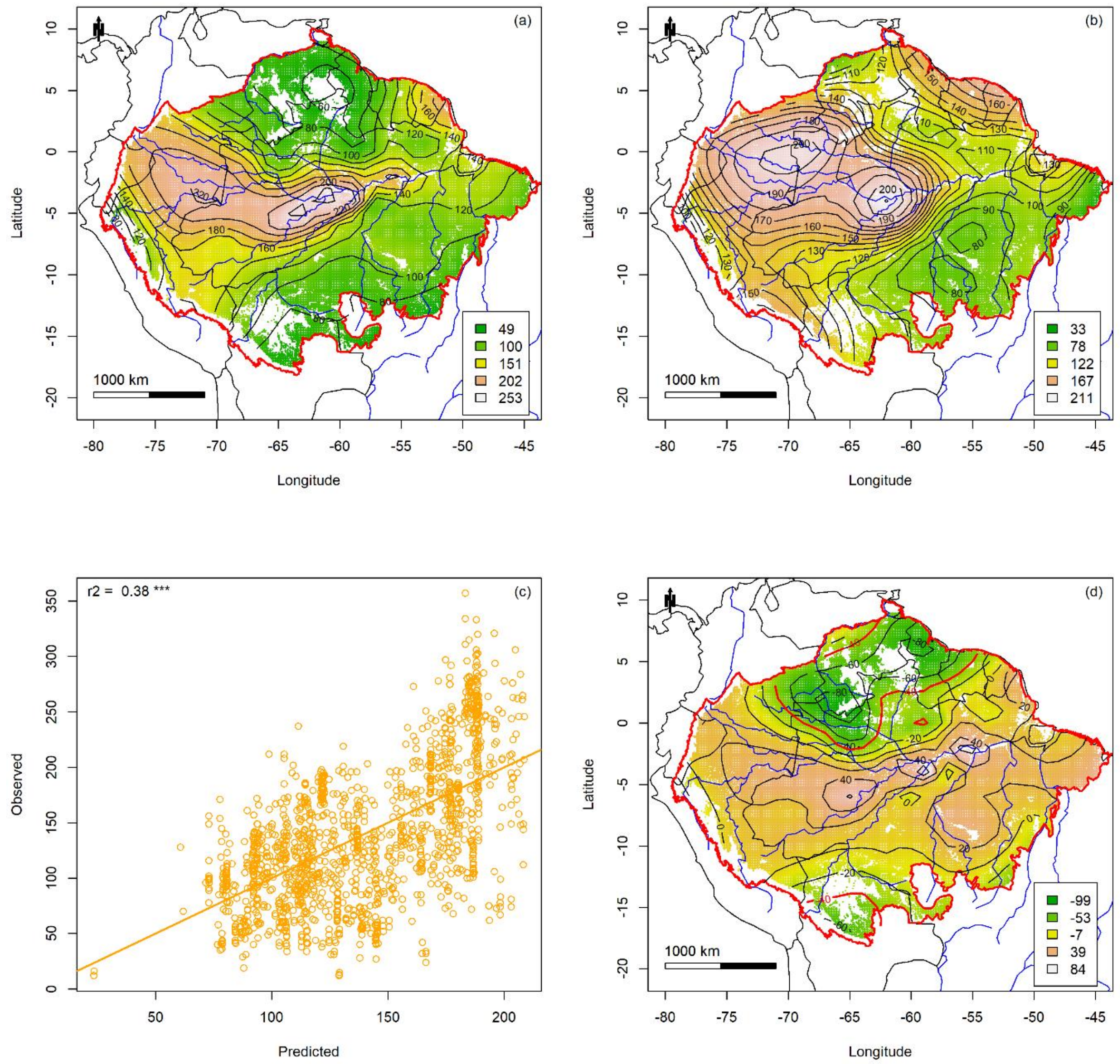

Supplementary Fig. 22. **The effect of cumulative water deficit (mm), regional tree-density, and collecting intensity on tree species-richness**. **A**) Tree species-richness observed. **B**) Tree species-richness as predicted by cumulative water deficit, regional tree-density, and collecting intensity. **C**) Model performance, showing predicted and observed tree species-richness. **D**) Residuals of tree species-richness predicted by cumulative water deficit, regional tree-density, and collecting intensity (A – B). Maps created with custom R (32) script. Red polygon: Amazonian Biome limit [2]. Maps created with a custom R [3] script. Base map source (country.shp, rivers.shp): ESRI (http://www.esri.com/data/basemaps, © Esri, DeLorme Publishing Company).

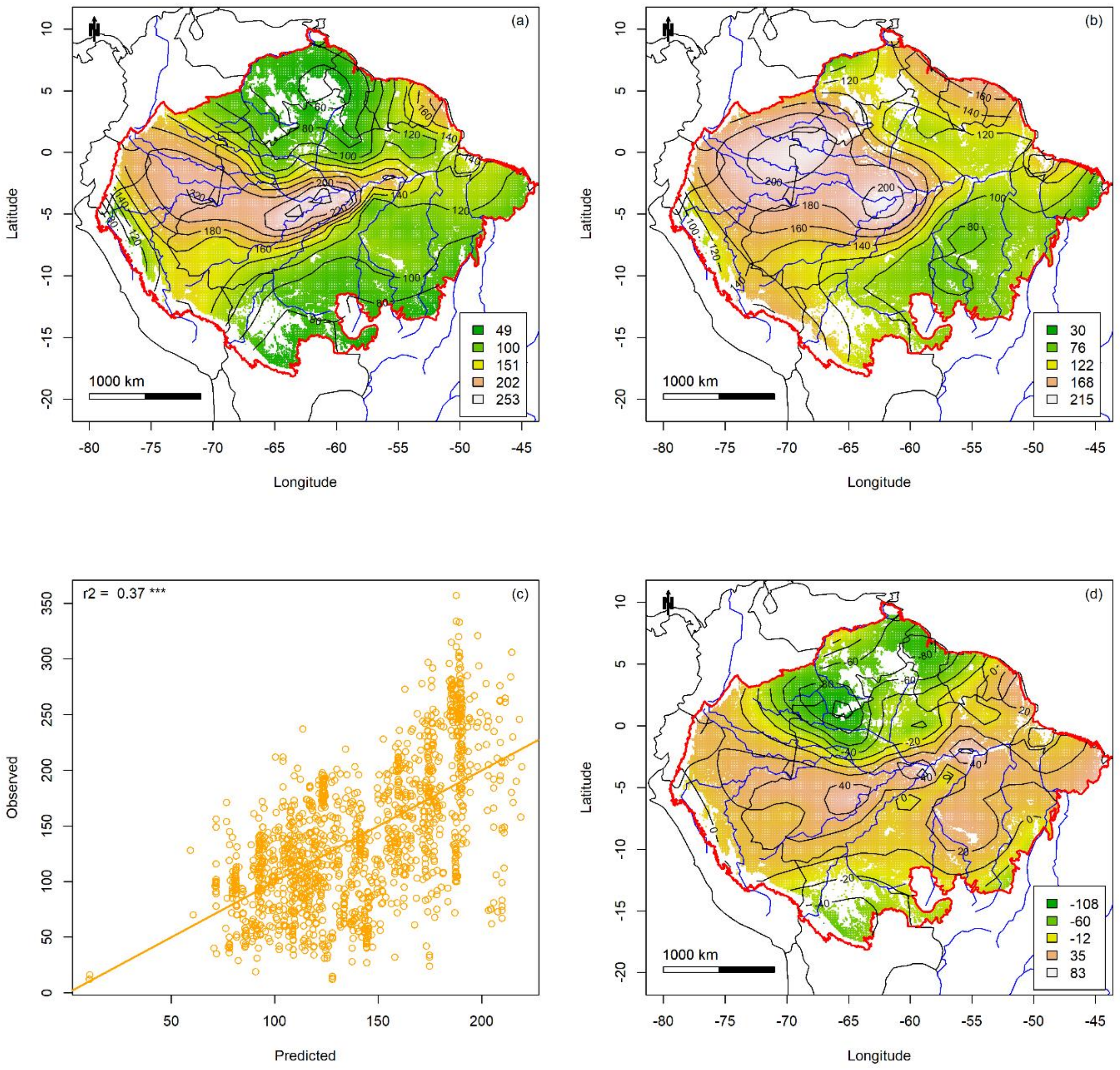

Supplementary Fig. 23. The effect of cumulative water deficit (mm), regional tree-density, and soil fertility (location on Pebas) on tree species-richness. **A**) Tree species-richness observed. **B**) Tree species-richness as predicted by cumulative water deficit, regional tree-density, and location on Pebas formation. **C**) Model performance, showing predicted and observed tree species-richness. **D**) Residuals of tree species-richness predicted by cumulative water deficit, regional tree-density, and location on Pebas formation (A – B). Maps created with custom R (32) script. Red polygon: Amazonian Biome limit [2]. Maps created with a custom R [3] script. Base map source (country.shp, rivers.shp): ESRI (http://www.esri.com/data/basemaps, © Esri, DeLorme Publishing Company).

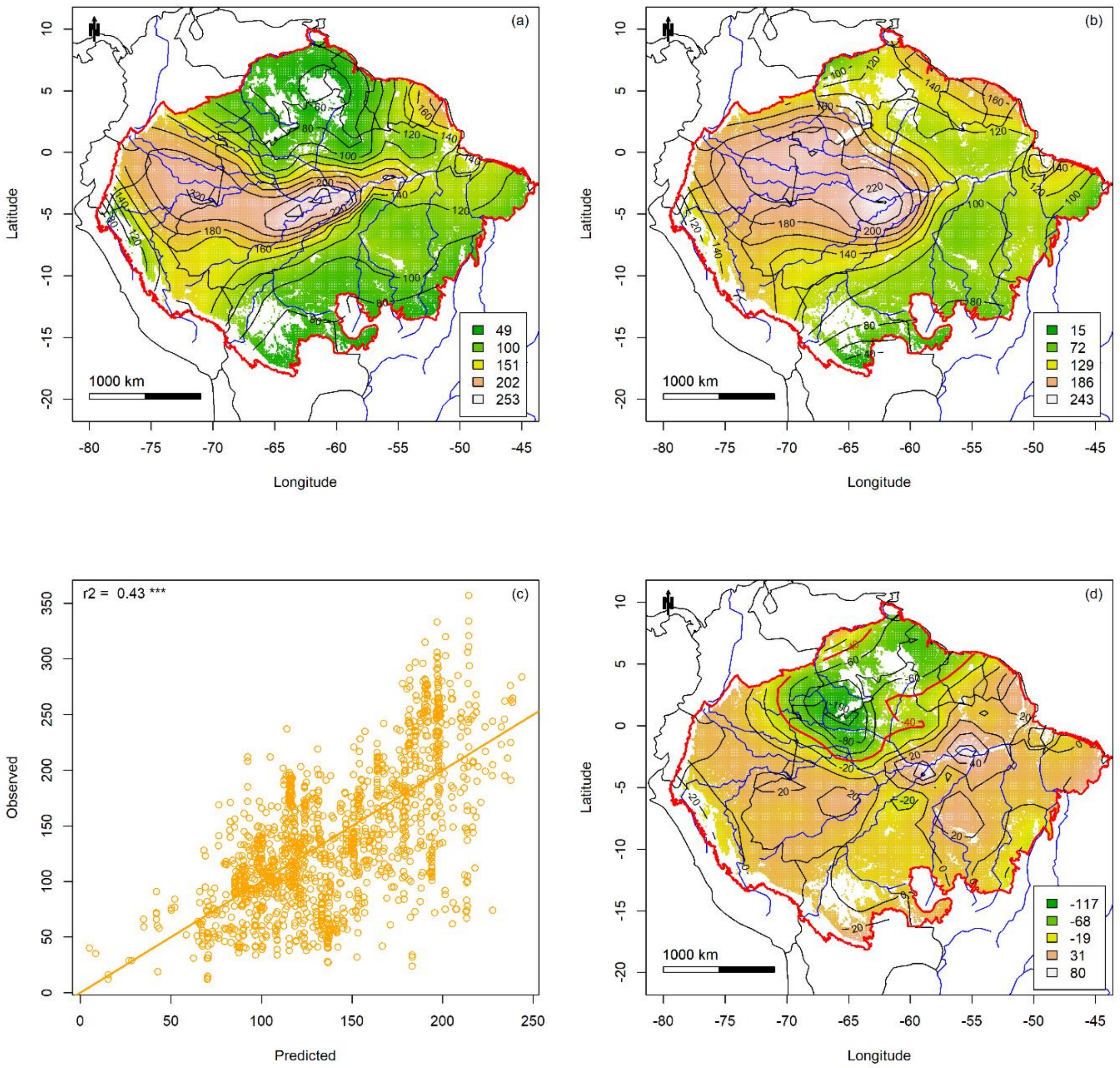

Supplementary Fig. 24. **The effect of cumulative water deficit (mm), regional tree-density, temperature seasonality, and collecting intensity on tree species-richness**. **A**) Tree species-richness observed. **B**) Tree species-richness as predicted by cumulative water deficit, regional tree-density, temperature seasonality, and collecting intensity. **C**) Model performance, showing predicted and observed tree species-richness. **D**) Residuals of tree species-richness predicted by cumulative water deficit, regional tree-density, temperature seasonality, and collecting intensity (A – B). Maps created with custom R (32) script. Red polygon: Amazonian Biome limit [2]. Maps created with a custom R [3] script. Base map source (country.shp, rivers.shp): ESRI (http://www.esri.com/data/basemaps, © Esri, DeLorme Publishing Company).

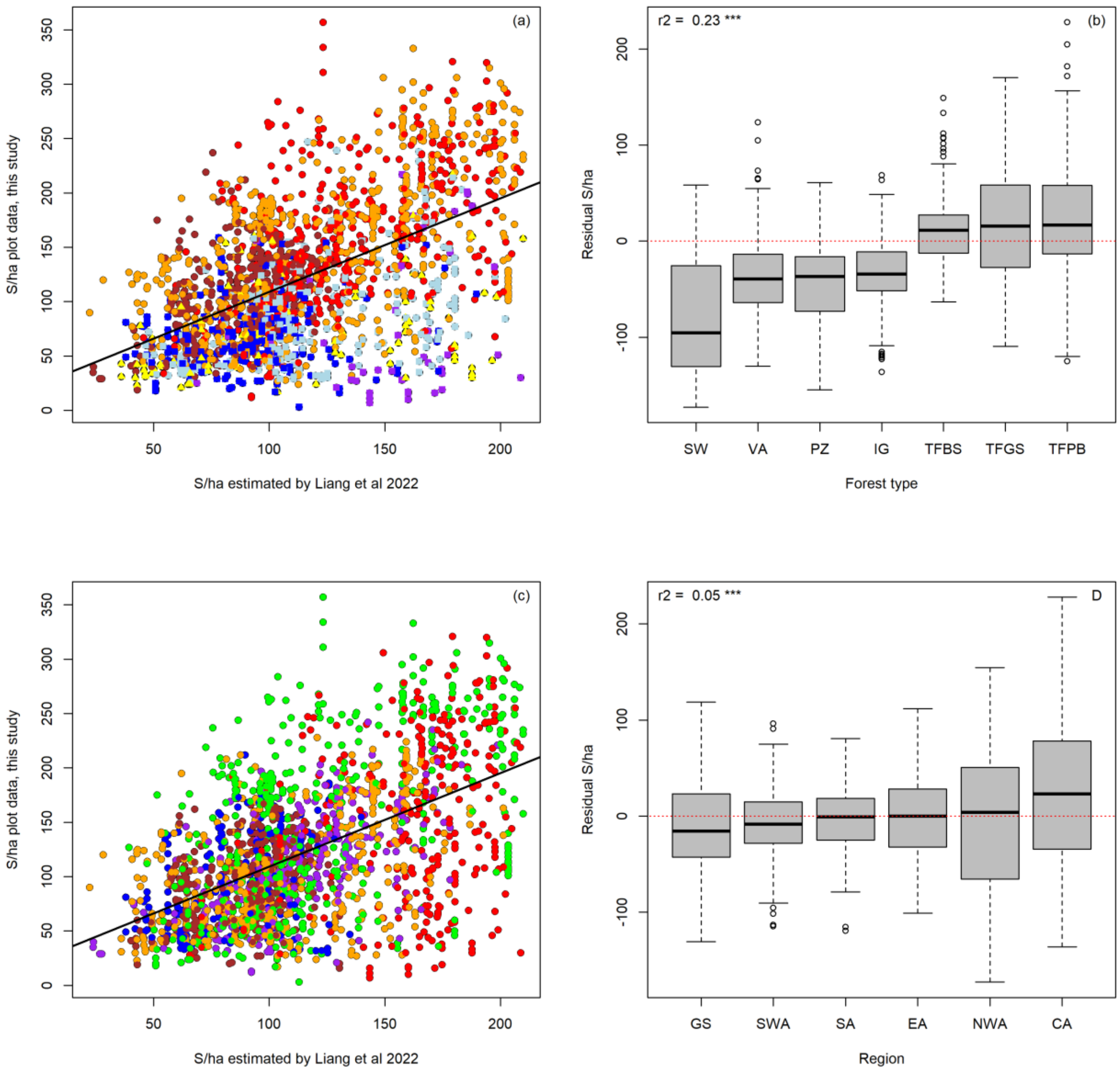

Supplementary Fig. 25. **Species richness as predicted by Liang et al (2022) A)** Species richness as estimated by Liang et al [7], as predictor for species richness on our 2046 plots (R^2 = 28%, P < 0.001). Red: Terra Firme Pebas Formation; Brown: Terra Firme Brazilian Shield; Orange: Terra Firme Guyana Shield; Yellow: White sand forest; Light blue: Varzea; Dark blue: Igapo; Purple: Swamp. **B)** Boxplot of residuals of the linear model of A. by forest type **C)** As A but legend by region: Orange: Guyana Shield; Blue: Eastern Amazonia; Green: Central Amazonia; Brown: Southern Amazonia; Purple: Southern West Amazonia; Red: North Western Amazonia. **D)** Boxplot of residuals of the linear model of C. by Amazonian region.

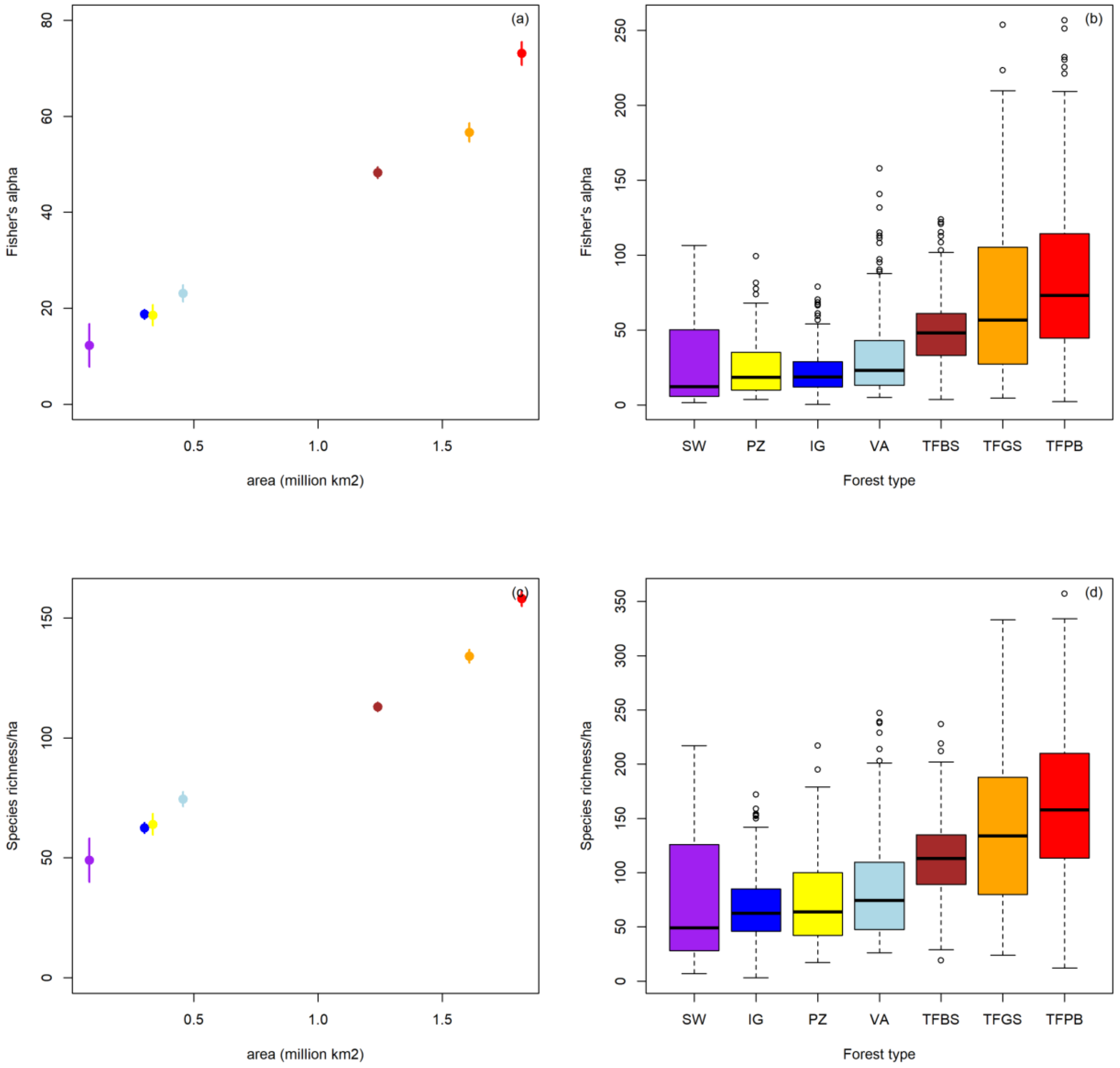

Supplementary Fig. 26 Area of major forest soil combination in Amazonia. **A)** Median tree alpha-diversity of seven main Amazonian forest types by surface area coverage of Amazonia. Bars indicate the standard error. **B)** Boxplot of tree alpha-diversity by main forest type. **C)** Median tree species-richness of seven main Amazonian forest types by surface area coverage of Amazonia. Bars indicate the standard error. **D)** Boxplot of tree species-richness by main forest type. Data for forest area in millions km^2 (SW, swamp forest, 0.0803 [8]; IG, igapó, 0.3024 [8]; VA, várzea forest, 0.4563 [8]; PZ, white sand forest, 0.335 [9]; TFGS, terra firme forest Guyana Shield [10,11], 1.61; TFBS, terra firme Brazilian Shield, 1.24 [11]; TFPB, terra firme Pebas formation, 1.82 [11]).

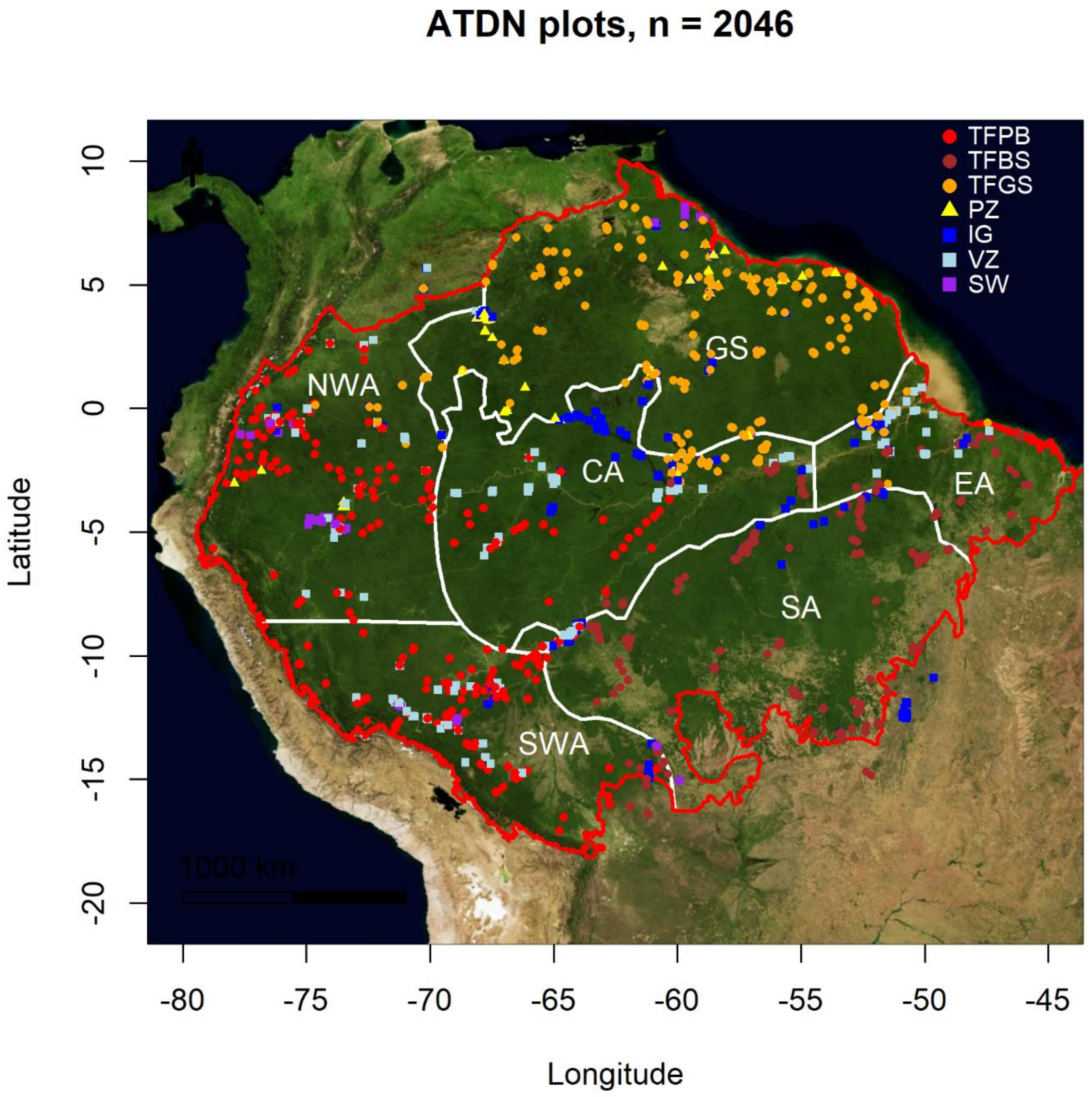

Supplementary Fig. 27 ATDN plots in Amazonia. Plots (2,046) of the Amazon Tree Diversity Network (ATDN) inventory data used for analyses (size between 0.5 and 2 ha). Amazonian Regions [12,13]: GS - Guyana Shield; CA – central Amazonia; EA – eastern Amazonia; SA – southern Amazonia; NWA north-western Amazonia; SWA – south-western Amazonia. Plot forest types: TFPB – terra-firme Pebas formation (415 plots); TFBS – terra-firme Brazilian Shield (386); TFGS – terra-firme Guyana Shield (641); PZ – forests on white sand (95); IG – igapó (222); VZ – várzea (240); SW – swamp forest (47). Plots outside the Amazonian polygon were used to improve interpolation near the edges. Red polygon: Amazonian Biome limit [2]. Map created with custom R[3] script. Background: Visible Earth NASA (https://visibleearth.nasa.gov/ © NASA).

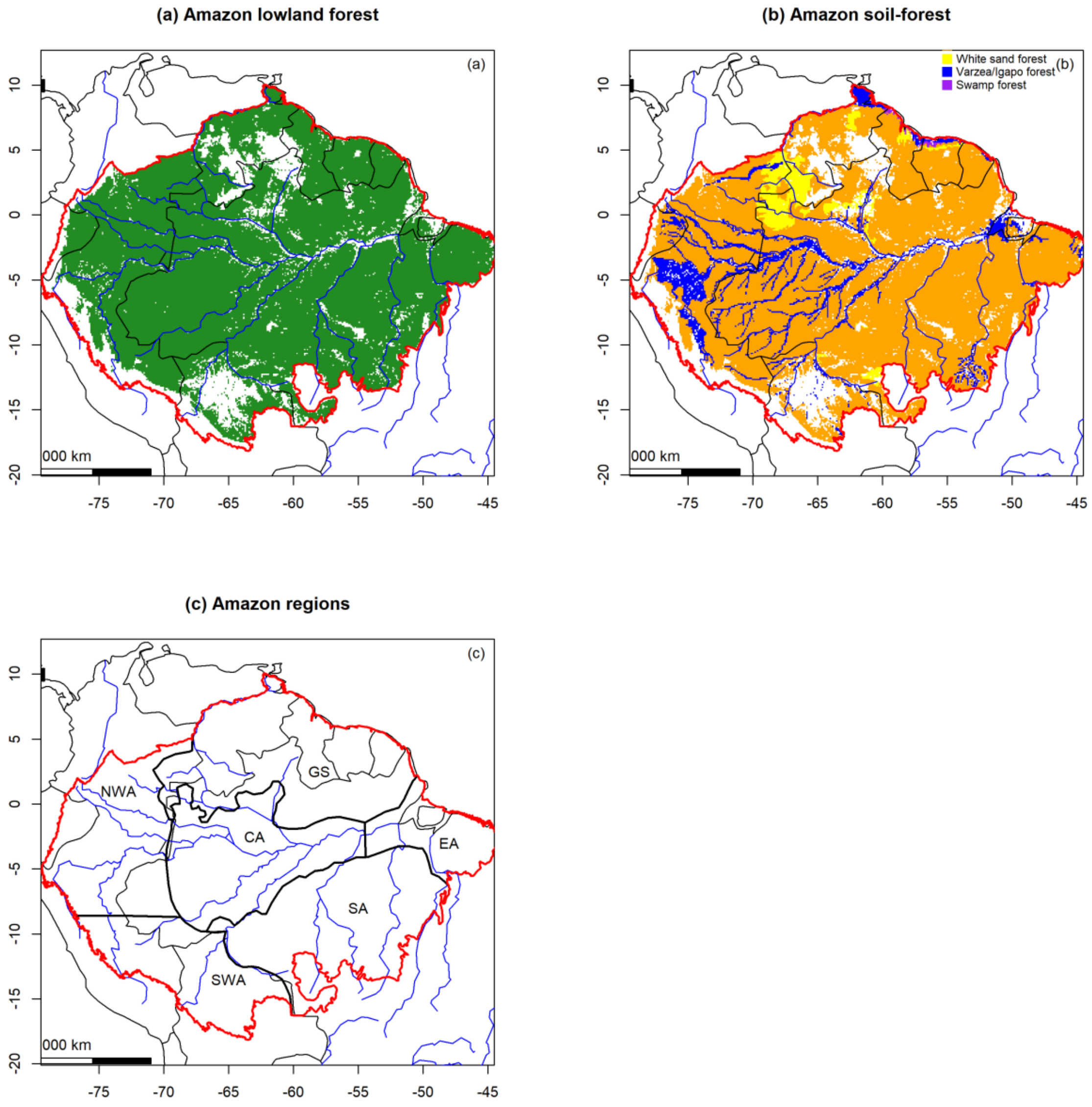

Supplementary Fig. 28 Amazonian base maps. A) The Amazonian lowland forest based on land area [14], minus large water bodies [14], minus areas above 500 m elevation [15], and minus areas originally without forest [16,17]. The originally forested area sums to approximately 5.79 million km^2. **B**) Major hydrology-soil-forest combinations of the Amazonian lowland forest with a major distinction in unflooded forests (terra-firme, white sand forest) and forest subject to long-term flooding (várzea, igapó) or waterlogging (swamps). Soils after [11,18]. **C**) Amazonian forest regions [12,13]: GS - Guyana Shield; CA – central Amazonia; EA – eastern Amazonia; SA – southern Amazonia; NWA north-western Amazonia; SWA – south-western Amazonia. Red polygon: Amazonian Biome limit [2]. Maps created with a custom R [3] script. Base map source (country.shp, rivers.shp): ESRI (http://www.esri.com/data/basemaps, © Esri, DeLorme Publishing Company).

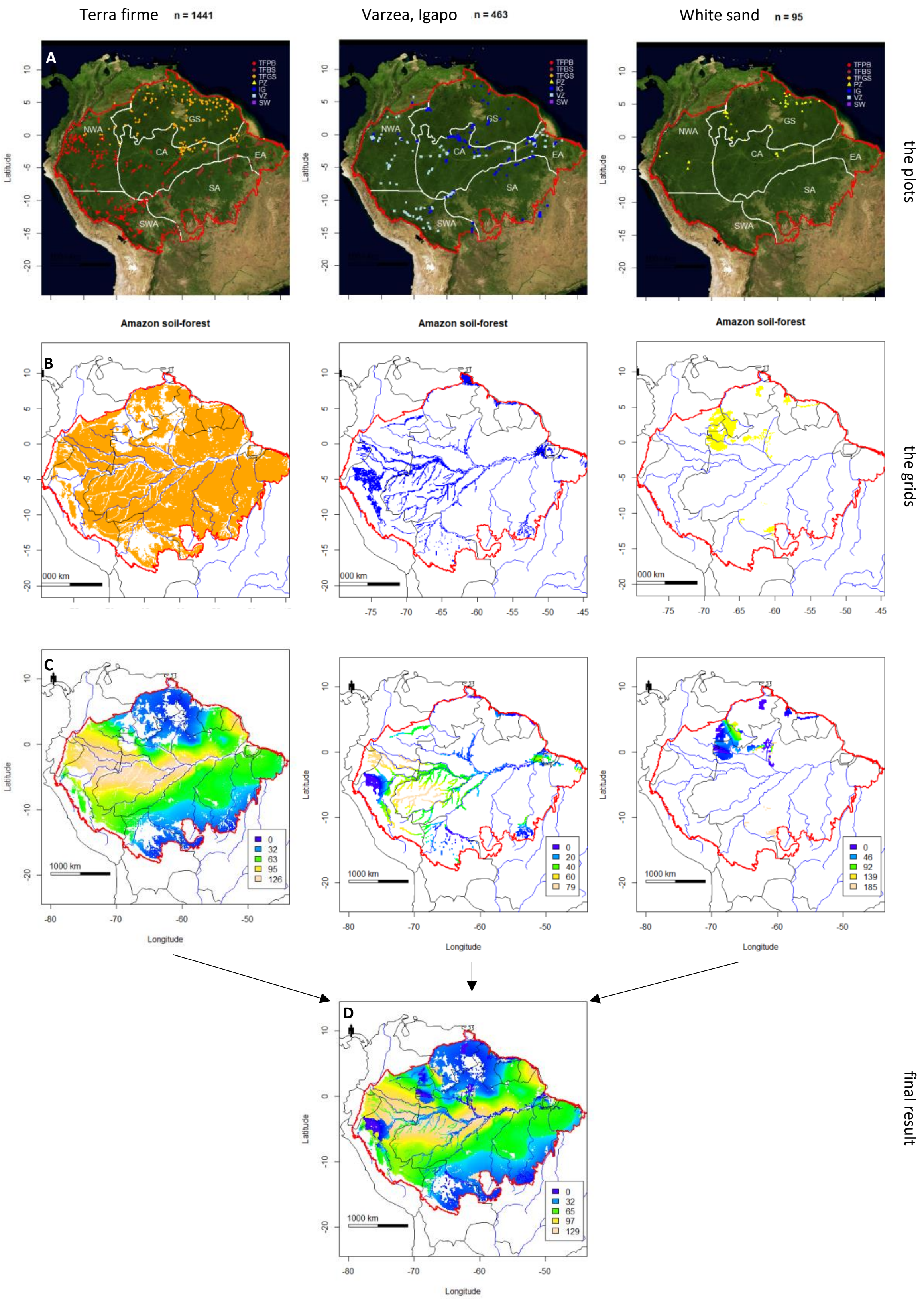

Supplementary Fig. 29. Model to predict Fisher's alpha for Amazonia at 0.1 degree. As example data from all plots on terra firme (**A**) was used to carry out a spatial loess regression (span 0.33). The resulting loess regression model was then predicted for grid cells classified as terra firme forest (orange in **B**) and mapped (**C**). This was done for terra-firme forests (left column), white sand forests (middle column), floodplain forests (right hand column), and swamp forest (not shown, due to space). All four results were then combined to create the final map (D). Red polygon: Amazonian Biome limit [2]. Maps created with a custom R [3] script. Base map source (country.shp, rivers.shp): ESRI (http://www.esri.com/data/basemaps, © Esri, DeLorme Publishing Company).

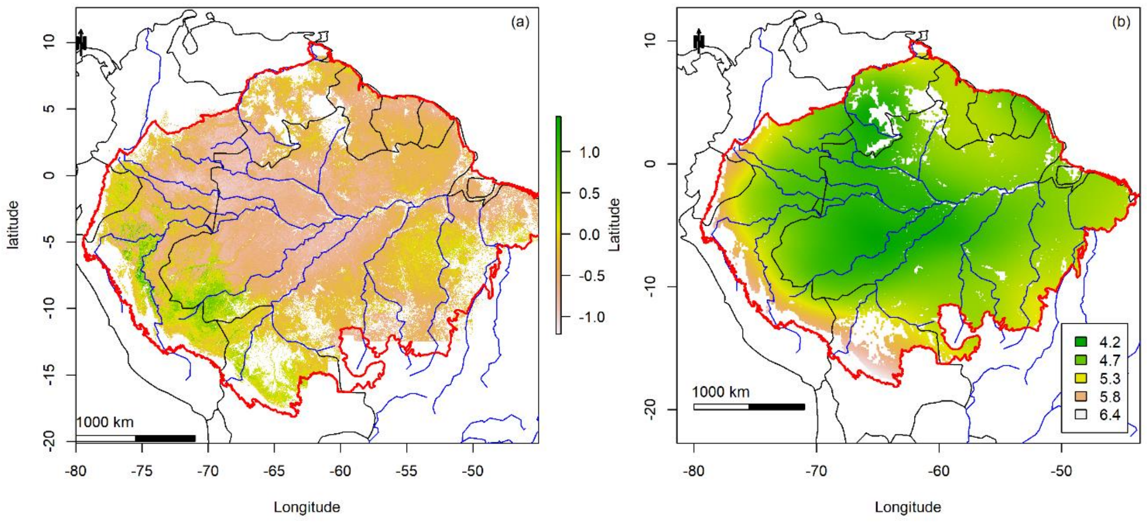

Supplementary Fig. 30. **Soil fertility A**) Sum of bases as $^{10}\log(K + Na + Ca + Mg)$ [19]. Higher sum of bases generally means higher fertility. **B**) pH (refs [6,20,21]), lower pH (more acid) generally means lower fertility. Red polygon: Amazonian Biome limit [2]. Maps created with a custom R [3] script. Base map source (country.shp, rivers.shp): ESRI (http://www.esri.com/data/basemaps, © Esri, DeLorme Publishing Company).

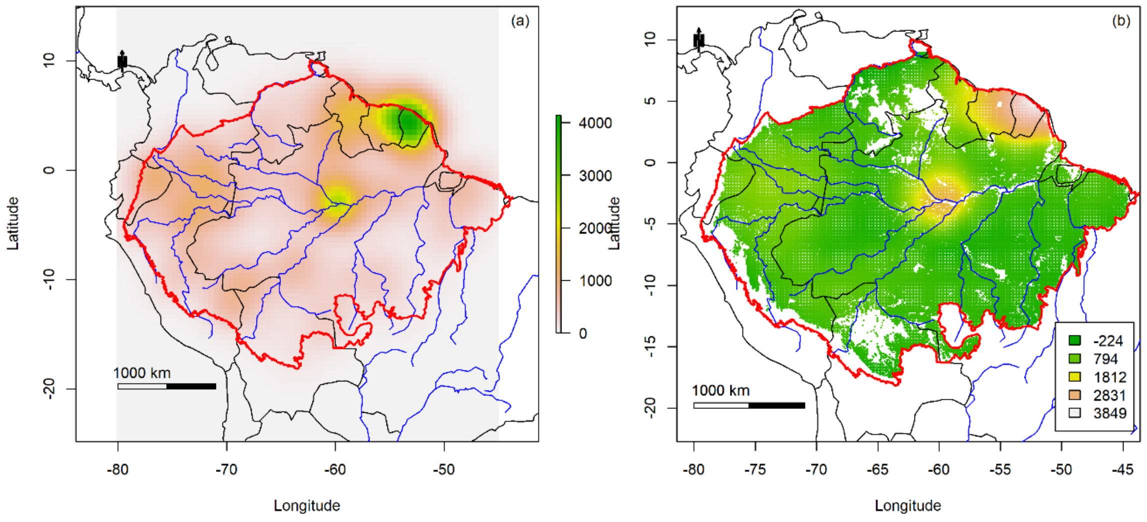

Supplementary Fig. 31. **Density of tree species collections in Amazonia. A**) Collecting density was based on the 530,025 unique collections of [22] using the standard Kernel density function of R with Gaussian smoothing and adjustment of 0.2. **B**) Collecting density predicted at plot level and interpolated with loess with span = 0.2. Red polygon: Amazonian Biome limit [2]. Maps created with a custom R [3] script. Base map source (country.shp, rivers.shp): ESRI (http://www.esri.com/data/basemaps, © Esri, DeLorme Publishing Company).

Table S1. Model performance based on the explained variation (R^2) in tree species-richness for various combinations of independent variables. The best models provide high explained variation with a low number of variables. The best model with three variables in indicated in bold and light grey, and is the model used as the 'best' model in figure 4.

**Dependent**	**tree density**	**Regional Dens.**	**Cum Water Def**	**Annual rainfall**	**Collecting dens.**	**Temp seasonality**	**pH**	**SB**	**Pebas**	**R^2 (%)**	**Figure**
Richness								x		0.4	S23
Richness							x			5	S22
Richness									x	5	S24
Richness					x					13	S25
Richness	x									16	S19
Richness				x						22	S21
Richness		x								26	S18
Richness			**x**							27	S20
Richness		x			x					29	-
Richness			x		x					29	-
Richness		x	x							37	-
Richness		x	x						x	37	S28
Richness		x	x		x					38	S27
**Richness**		**x**	**x**			**x**				**43**	**4**
Richness		x	x		x	x				43	S29

Supplementary References

1 ape: Analyis of phylogenetics and evolution (https://cran.r-project.org/web/packages/ape/index.html, 2021).

2 RAISG. (https://www.amazoniasocioambiental.org/en/, 2020).

3 R Development Core Team. R: A language and environment for statistical computing. Report No. ISBN 3-900051-07-0, (R Foundation for Statistical Computing, Vienna, Austria, 2017).

4 Stropp, J., ter Steege, H., Malhi, Y., ATDN & RAINFOR. Disentangling regional and local tree diversity in the Amazon. *Ecography* **32**, 46-54 (2009).

5 ter Steege, H., ATDN & RAINFOR. in *Amazonia: Landscape and Species Evolution* (eds C. Hoorn & F Wesselingh) Ch. 21, 349-359 (Wiley-Blackwell, 2010).

6 Zuquim, G. *et al.* Making the most of scarce data: Mapping soil gradients in data-poor areas using species occurrence records. *Methods in Ecology and Evolution* **10**, 788-801, doi:https://doi.org/10.1111/2041-210X.13178 (2019).

7 Liang, J. *et al.* Co-limitation towards lower latitudes shapes global forest diversity gradients. *Nature Ecology & Evolution*, doi:10.1038/s41559-022-01831-x (2022).

8 Wittmann, F. & Junk, W. in *The Wetland Book* (eds C. Finlayson, G. Milton, R. Prentice, & N. Davidson) 2-16 (Springer, 2016).

9 Adeney, J. M., Christensen, N. L., Vicentini, A. & Cohn-Haft, M. White-sand Ecosystems in Amazonia. *Biotropica* **48**, 7-23, doi:https://doi.org/10.1111/btp.12293 (2016).

10 Hammond, D. S. in *Tropical forests of the Guiana Shield : ancient forests in a modern world* (ed D. S. Hammond) 1-14 (CABI Pub., Wallingford, 2005).

11 Quesada, C. A. *et al.* Soils of Amazonia with particular reference to the RAINFOR sites. *Biogeosciences* **8**, 1415-1440, doi:10.5194/bg-8-1415-2011 (2011).

12 ter Steege, H. *et al.* Hyperdominance in the Amazonian tree flora. *Science* **342**, 1243092, doi:10.1126/science.1243092 (2013).

13 Feldpausch, T. R. *et al.* Tree height integrated into pantropical forest biomass estimates. *Biogeosciences* **9**, 3381-3403, doi:10.5194/bg-9-3381-2012 (2012).

14 Environmental Systems Research Institute. ESRI Data & Maps 1999 - An ESRI White Paper. (Environmental Systems Research Institute, Redlands, US, 1999).

15 Shuttle Radar Propulsion Mission. NASA Jet Propulsion Laboratory. <http://www2.jpl.nasa.gov/srtm/> (2009).

16 Soares-Filho, B. S. *et al.* Modelling conservation in the Amazon basin. *Nature* **440**, 520-523, doi:http://www.nature.com/nature/journal/v440/n7083/suppinfo/nature04389_S1.html (2006).

17 Soares-Filho, B. S. *et al.* (http://daac.ornl.gov//LBA/guides/LC14_Amazon_Scenarios.html, 2013).

18 Dijkshoorn, J. A., Huting, J. R. M. & Tempel, P. Update of the 1:5 million Soil and Terrain Database for Latin America and the Caribbean (SOTERLAC; version 2.0). (ISRIC - World Soil Information, Wageningen, 2005).

19 Zuquim, G. *et al.* Introducing a map of soil base cation concentration, an ecologically relevant GIS-layer for Amazonian forests. *Geoderma Regional* **33**, e00645, doi:https://doi.org/10.1016/j.geodrs.2023.e00645 (2023).

20 Poels, R. L. H. *Soils water and nutrients in a forest ecosystem in Surinam* PhD thesis, Wageningen University, (1987).

21 van Kekem, A. J., Pulles, J. H. M. & Khan, Z. *Soils of the Rainforest in Central Guyana*. Vol. 2 (Tropenbos Guyana Programme, 1996).

22 ter Steege, H. *et al.* The discovery of the Amazonian tree flora with an updated checklist of all known tree taxa. *Scientific Reports* **6**, 29549, doi:10.1038/srep29549 (2016).
